# Prolonged experimental drought reduces plant hydraulic conductance and transpiration and increases mortality in a piñon–juniper woodland

**DOI:** 10.1002/ece3.1422

**Published:** 2015-03-23

**Authors:** Robert E Pangle, Jean-Marc Limousin, Jennifer A Plaut, Enrico A Yepez, Patrick J Hudson, Amanda L Boutz, Nathan Gehres, William T Pockman, Nate G McDowell

**Affiliations:** 1 Department of Biology, MSC03 2020, 1 University of New MexicoAlbuquerque, New Mexico, 87131-0001; 2 Centre d'Ecologie Fonctionnelle et Evolutive CEFE, UMR5175, CNRS, Université de Montpellier, Université Paul-Valéry Montpellier, EPHE1919 Route de Mende, Montpellier Cedex 5, 34293, France; 3 Departamento de Ciencias del Agua y del Medio Ambiente, Instituto Tecnológico de SonoraCiudad Obregón, Sonora, 85000, Mexico; 4 Earth and Environmental Sciences Division, Los Alamos National LaboratoryLos Alamos, New Mexico, 87545

**Keywords:** Canopy dieback, climate change, hydraulic failure, net carbon assimilation, plant water stress, precipitation manipulation, stomatal response to drought, tree death

## Abstract

Plant hydraulic conductance (*k*_s_) is a critical control on whole-plant water use and carbon uptake and, during drought, influences whether plants survive or die. To assess long-term physiological and hydraulic responses of mature trees to water availability, we manipulated ecosystem-scale water availability from 2007 to 2013 in a piñon pine (*Pinus edulis*) and juniper (*Juniperus monosperma*) woodland. We examined the relationship between *k*_s_ and subsequent mortality using more than 5 years of physiological observations, and the subsequent impact of reduced hydraulic function and mortality on total woody canopy transpiration (*E*_C_) and conductance (*G*_C_). For both species, we observed significant reductions in plant transpiration (*E*) and *k*_s_ under experimentally imposed drought. Conversely, supplemental water additions increased *E* and *k*_s_ in both species. Interestingly, both species exhibited similar declines in *k*_s_ under the imposed drought conditions, despite their differing stomatal responses and mortality patterns during drought. Reduced whole-plant *k*_s_ also reduced carbon assimilation in both species, as leaf-level stomatal conductance (*g*_s_) and net photosynthesis (*A*_n_) declined strongly with decreasing *k*_s_. Finally, we observed that chronically low whole-plant *k*_s_ was associated with greater canopy dieback and mortality for both piñon and juniper and that subsequent reductions in woody canopy biomass due to mortality had a significant impact on both daily and annual canopy *E*_C_ and *G*_C_. Our data indicate that significant reductions in *k*_s_ precede drought-related tree mortality events in this system, and the consequence is a significant reduction in canopy gas exchange and carbon fixation. Our results suggest that reductions in productivity and woody plant cover in piñon–juniper woodlands can be expected due to reduced plant hydraulic conductance and increased mortality of both piñon pine and juniper under anticipated future conditions of more frequent and persistent regional drought in the southwestern United States.

## Introduction

Global analyses suggest an increased frequency of drought-induced forest and woodland tree mortality (Allen et al. 2010; Carnicer et al. 2011; Peng et al. 2011; Das et al. 2013). Drought-related tree mortality has far reaching implications for a number of important ecosystem-related services – ranging from significant reductions in carbon uptake and carbon sequestration in impacted areas to alterations in eco-hydrological function (Huang et al. 2010; Adams et al. 2012; Huang and Anderegg 2012). Uncertainty about how changes in climate and precipitation patterns will impact vegetation has motivated efforts to more fully understand the physiological mechanisms that contribute to and cause tree mortality, and utilizing these crucial insights, refine mortality routines in models such as dynamic global vegetation models (DGVM) to better predict terrestrial vegetation responses to anticipated climate change, both at the local and global scale (Sitch et al. 2008; Fisher et al. 2010; McDowell et al. 2011, 2013; Jiang et al. 2013; Xu et al. 2013; Zeppel et al. 2013). The southwestern region of the United States (SWUS) provides an excellent case study to examine drought-related tree mortality because of the well-documented pattern of regional-scale dieback events (Allen and Breshears 1998; Breshears et al. 2005; Koepke et al. 2010; Ganey and Vojta 2011; Bowker et al. 2012; Clifford et al. 2013; Kukowski et al. 2013). Recent modeling efforts utilizing long-term tree ring records and estimates of future drought severity (Seager et al. 2007; Dominguez et al. 2010) suggest significant, or even complete, conifer mortality in some parts of the SWUS by 2050 (Jiang et al. 2013; Williams et al. 2013). Within the SWUS, extensive tree mortality has already been observed in semiarid piñon–juniper woodlands in recent decades (see Breshears et al. 2005), a cover type that dominates a significant area within the region (Romme et al. 2009). Here, we focus on the long-term physiological responses of mature piñon (*Pinus edulis*) and juniper (*Juniperus monosperma*) trees to experimentally imposed and naturally occurring drought, and the co-occurring consequences of reduced physiological function and subsequent tree mortality on canopy processes at the ecosystem scale. Piñon–juniper is an excellent system to study long-term physiological responses to imposed drought because these two species exhibit differing plant hydraulic response strategies to water stress.

Two categories of physiological response have been theorized to occur in regard to drought-induced tree mortality: (1) hydraulic failure due to tissue desiccation and/or xylem cavitation, or (2) carbon limitation resulting from stomatal closure and lack of carbon assimilation, transport, and utilization during prolonged drought (McDowell et al. 2008, 2011; Sala et al. 2010; McDowell 2011). Plants generally close stomata to limit excessive water loss during periods of high evaporative demand, especially during drought periods of limited soil water (Sperry 2000; McDowell et al. 2008). Closing stomata prevents or limits xylem cavitation and hydraulic failure in trees, but necessarily limits CO_2_ uptake (Sperry 2000; Maherali et al. 2006). Trees generally respond to declining soil and foliar water potentials with stomatal responses that fall along a continuum ranging from anisohydric to strictly isohydric stomatal behavior (Tardieu and Simonneau 1998; but see Franks et al. 2007 and Klein 2014 for critical discussions related to this dichotomy). Relatively anisohydric trees, such as juniper, generally do not fully close stomata due to declining soil water potential (*Ψ*_S_) during drought, instead, they continue foliar gas exchange and allow their foliar and xylem water potential (*Ψ*_L_ and *Ψ*_XY_) to decrease as *Ψ*_S_ declines (West et al. 2008; Plaut et al. 2012). In contrast, species exhibiting more isohydric behavior, such as piñon pine, close their stomata to prevent *Ψ*_L_, and thus *Ψ*_XY_ from falling below some threshold associated with significant xylem cavitation and loss of hydraulic function (West et al. 2008; Plaut et al. 2012). These response strategies have differing implications for trees depending on the severity and duration of drought events and the vulnerability to xylem cavitation of the species (McDowell et al. 2008, 2011). Relatively isohydric species have been hypothesized to be potentially more prone to carbon limitation, as they close stomata early in drought, thus limiting carbon assimilation while respiration continues to consume carbon reserves, whereas anisohydric species have been hypothesized to be more prone to hydraulic failure (McDowell et al. 2008; Breshears et al. 2009).

Recently, experimental studies have attempted to examine which type of physiological response dominates in dying trees, that is, hydraulic failure or carbon limitation (Adams et al. 2009, 2013; Anderegg et al. 2012; Plaut et al. 2012; Anderegg and Anderegg 2013; Hartmann et al. 2013a,b; Mitchell et al. 2013; Dickman et al. 2014; Sevanto et al. 2014). While hydraulic failure has been implicated in some instances of drought-related tree mortality (see Anderegg et al. 2012; Anderegg and Anderegg 2013; Hartmann et al. 2013a; Mitchell et al. 2013), declines in foliar nonstructural carbohydrates (NSC) have been reported in instances where experimentally induced drought and subsequent tree mortality were observed (Adams et al. 2009, 2013; Mitchell et al. 2013; Dickman et al. 2014). In other cases, mortality could not be attributed to either hydraulic failure or carbon limitation (Hartmann et al. 2013b; Sevanto et al. 2014), as these processes do not necessarily occur independently or exclusively of each other in drought-stricken trees (McDowell 2011; McDowell et al. 2011; Hartmann et al. 2013b; Dickman et al. 2014).

Whole-plant hydraulic conductance (*k*_s_; per unit sapwood area; mol m^−2^ s^−1 ^MPa^−1^) is a key component of plant drought response (McDowell et al. 2008). Specifically, *k*_s_ provides a measure of the hydraulic capacity of xylem tissue to supply water to the canopy to replace transpiration (*E*) losses during the process of CO_2_ uptake (Sperry et al. 1998; Whitehead 1998; Sperry 2000). The reduction/loss of *k*_s_ during drought can occur at multiple points in the soil-to-leaf hydraulic pathway, including the soil-to-root interface, and within root, stem, and canopy xylem tissues (Sperry et al. 1998). The recovery of *k*_s_ after loss during drought depends first on water availability and secondarily on the plant's ability (or lack thereof) to refill embolized xylem conduits and/or grow new xylem tissue (Hacke and Sperry 2003; Cochard and Delzon 2013; Meinzer and McCulloh 2013; Wheeler et al. 2013; Klein et al. 2014). A permanent reduction in *k*_s_ can have various consequences for plants, ranging from outright desiccation (and death) during drought to a reduced ability to transpire water and assimilate carbon, even during periods when water is not limiting (Brodribb and Cochard 2009). In either case, the risk of hydraulic failure and potential carbon limitation increases for plants with low or severely reduced hydraulic conductance.

To assess the long-term physiological and hydraulic responses to altered water availability, we implemented a long-term study that experimentally modified water availability in adult piñon and juniper trees growing in a natural setting (Pangle et al. 2012). Long-term studies that continuously measure the physiological and hydraulic response of trees to experimentally imposed drought are not common, and thus, this study provides a unique data set to assess the relationship between tree hydraulic function and subsequent mortality events due to imposed water stress. Our in situ experimental manipulations were initiated in 2007, and subsequently, we measured various physiological responses, tree canopy dieback, and tree mortality through 2012. For this analysis, we report on the response of whole-plant *k*_s_ and *E* to long-term changes in precipitation and examine the relationship between reduced *k*_s_ and susceptibility to drought-induced mortality for both piñon and juniper. We hypothesized that experimentally imposed drought would increase plant water stress, and reduce *k*_s_ and *E* in both species and that these reductions would predispose droughted trees to increased mortality and/or canopy dieback. Conversely, we hypothesized that increased precipitation via supplemental irrigation would increase both *E* and whole-plant *k*_s_ relative to both droughted and ambient trees. Finally, we sought to quantify the combined impacts of variable precipitation, reduced hydraulic conductance, and subsequent mortality and aboveground biomass reductions on total woody canopy transpiration (*E*_C_) and canopy conductance (*G*_C_) at both daily and annual timescales.

## Methods

### Study site and experimental plots

This research was conducted at the Sevilleta National Wildlife Refuge in central New Mexico, in experimental plots established on the eastern slope of the Los Pinos Mountains (34°23′11′' N, 106°31′46′' W, 1911 m). The site is a piñon pine (*Pinus edulis,* Engelm.) and juniper (*Juniperus monosperma,* (Engelm.) Sarg.) woodland, with piñon and juniper basal area and canopy coverage that averaged 20.0 m^2^ ha^−1^ and 36.7%, respectively, across the study site. Climate records (20 years, 1989–2009) from a nearby LTER meteorological station (Cerro Montosa #42; http://sev.lternet.edu/) indicate mean annual precipitation of 362.7 mm year^−1^. The region is strongly influenced by the North American Monsoon, with a large fraction of annual precipitation occurring in July, August, and September. Mean annual temperature (20 years) at this nearby LTER site was 12.7°C, with a mean July maximum of 31.0°C and a mean December minimum of −3.3°C. In total, the study site consisted of 12 experimental plots located in three replicate blocks that varied in slope percentage, aspect, and soil depth. A more detailed description and discussion of the vegetative cover and soil properties at this site have been presented elsewhere (see Pangle et al. 2012; Plaut et al. 2012).

The study utilized four different experimental treatments applied in three replicate blocks. The four experimental treatments included (1) unmanipulated, ambient control plots, (2) drought plots, (3) supplemental irrigation plots, and (4) cover-control plots. The three replicated blocks differed in their slope and aspect. One block was located on south-facing slopes, one on north-facing slopes, and one in a flat area of the landscape. Drought, cover-control, and irrigation infrastructure was installed in 2007, with drought treatments effectively in place by August 2007. The irrigation system was tested in 2007, and supplemental irrigations began in 2008.

### Experimental treatment design

To effectively reduce water availability to trees, we constructed three replicated drought structures that covered an area of 40 × 40 m (1600 m^2^) each. Each drought plot consisted of 29 parallel troughs running across the 40-m plot, constructed with thermoplastic polymer sheets fixed to horizontal rails that were approximately 1 m in height. The plastic sheets were bent into a concave shape to collect and divert the precipitation off-plot. The total plastic coverage in each plot was ∼45% (±1%) of the 1600-m^2^ plot area, resulting in ∼55% of ambient precipitation reaching the ground in drought plots. Plaut et al. (2013) compare the severity of these treatments to historical drought conditions observed in the past 100 years.

In addition, we built cover-control infrastructures to assess the impact of the plastic drought structures independent of reduced precipitation. The cover-control plots were the same size as drought plots, but with plastic sheets arranged in a convex orientation, which allowed precipitation to reach the plot surface rather than being diverted off-plot. Our irrigation system consisted of above-canopy sprinkler nozzles configured to deliver a supplemental rainstorm event of 19 mm (∼rate of 19 mm h^−1^). To avoid unintended manipulation of soil ionic composition, irrigation water was generated via reverse osmosis from Albuquerque NM municipal water, transported to the site, and stored in above ground tanks prior to application. Supplemental irrigations (19 mm event^−1^) were applied throughout the growing season (∼ monthly intervals) at an annual rate of 57, 69.5, 112, 107, and 95 mm year^−1^ from years 2008 through 2012.

### Site abiotic monitoring

We used Campbell Scientific dataloggers (CR1000, CR10X, and CR7) and a solar-powered wireless network (utilizing NL100 relays, Campbell Scientific, Logan, UT) to continuously monitor and record abiotic conditions and physiological measurements across the site (see Pangle et al. 2012). The south-facing replicate block of experimental plots was extensively instrumented with sensors to measure both abiotic and plant physiological parameters in 2007. The experimental plots in the north facing and flat blocks were initially only instrumented to monitor abiotic conditions in 2007, and sensors to measure plant physiological parameters (i.e., stem sap-flow) were added to these replicate blocks in 2009.

In all plots, soil volumetric water content (VWC, at 5 cm depth and at *n *=* *3 deeper depths) was measured beneath both piñon and juniper canopies, and in open intercanopy areas (with VWC measured via model EC-20 and EC-5 soil moisture probes, Decagon, Pullman, WA). In one of our replicate blocks, we measured soil water potential (*Ψ*_S_) at three depths using PST-55 psychrometers (Wescor, Inc., Logan, UT) and CR-7 dataloggers. An additional CR-10X datalogger was used to record data from a micrometeorological tower centrally located in an open intercanopy area of the study site. This meteorological tower recorded precipitation, photosynthetically active radiation (PAR), wind speed and direction, air temperature, and relative humidity (RH %*)*. During winter months, the rain gauge was fitted with a snow adaptor to thaw snow and record the total amount of liquid precipitation (in mm). All meteorological measurements were made at a height of 1–3 m above ground depending on the sensor array in question. For a more detailed and extensive description of the experimental design, our precipitation manipulation infrastructure, and the instrumentation and sensor scheme employed to monitor site abiotic parameters (please see Pangle et al. (2012) and Plaut et al. (2012)).

### Plant physiological responses

Multiple physiological characteristics of ten intensively monitored trees (five piñon and five juniper) within each plot were continually assessed by automated sensors and periodic field measurements to evaluate tree responses to precipitation manipulations. In addition, we performed periodic assessments of canopy greenness and canopy dieback (for the same trees) during each growing season to assess mortality response to precipitation treatments. Across all replicate blocks, a total of *n *=* *120 trees (*n *=* *60 per species) were initially designated and monitored across the 5+ year duration of the study. For each treatment factor, a subset of *n *=* *30 intensively monitored trees (*n *=* *15 per species) were subjected to each level of precipitation manipulation (i.e., ambient, drought, supplemental irrigation, and cover-control treatment). When available within a given experimental plot, extra replacement trees were designated and monitored in place of any of the original *n *=* *10 trees that experienced either mortality or severe canopy dieback over the 5+ year duration of the experiment, and the utilization of replacement trees within plots allowed us to continue the assessment of physiological and mortality responses to our long-term precipitation manipulations.

Predawn (*Ψ*_PD_) and midday (*Ψ*_MD_) plant (foliar) water potentials were measured with multiple Scholander-type pressure chambers (PMS Instrument Co, Albany, OR) on excised twigs from monitored trees (*n *=* *10 per plot) throughout the growing season of each year. Additionally, foliar *Ψ*_PD_ and *Ψ*_MD_ were measured both before and after supplemental irrigation events in irrigated and ambient plots to assess irrigation responses (with ambient plots serving as controls).

In order to assess *E* and *k*_s_, stem sap-flow (*J*_S_) was measured in monitored trees (*n *=* *10 per plot) using Granier heat dissipation sap-flow sensors installed in 2007 in each plot within the south aspect block (plots 9–12). Trees in north facing (plots 5–8) and flat blocks (plots 1–4) were instrumented with sap-flow sensors during the 2009 season, and the south-facing block was re-instrumented with new replacement sap-flow sensors in 2010. All monitored trees had two 10-mm Granier sap-flow sensors installed in the outermost sapwood (Granier 1987). Each sensor used the traditional two-probe heated and unheated reference design (Granier 1987), with two additional probes located 5 cm to the right side of the primary probes to correct for axial temperature gradients in the stem (see Goulden and Field 1994). This compensation for axial temperature gradients is critical to reduce measurement noise resulting from the open canopy and high-radiation environment of this ecosystem. In addition, stems were wrapped with reflective insulation (Reflectix Inc., Markleville, IN) to minimize short-term ambient temperature fluctuations and direct solar irradiance around probe installations. Sap-flow (*J*_S_) was calculated according to the methods outlined in Granier (1987) and Goulden and Field (1994). Sapwood depth was greater than 10 mm on the majority of instrumented trees; thus, only a very small percentage of measurements across the 2007–2012 period required a correction due to sensor installation in nonfunctional stem heartwood (see Clearwater et al. 1999). All data from sap-flow sensors were recorded using Campbell Scientific AM16/32 multiplexers and CR1000 dataloggers (Campbell Scientific, Logan, UT). Data processing was performed using Matlab software (R2011a; The Mathworks, Natick, MA).

Errors were minimized when employing Granier type sap-flow probes. First, the calculation of sap-flux density using the empirical relationship developed by Granier (1987) requires that a measurement of the maximum daily temperature difference between heated and unheated reference probes (i.e., “Δ*T*_max_”) be made under conditions of zero water flux in the stem (which generally occurs at night, when stomata are closed and vapor pressure deficit (VPD) is low). We made measurements of nocturnal stomatal conductance (*g*_s_) and *E* using a LI-1600 porometer (LI-COR, Inc., Lincoln, NE) under both premonsoon and monsoon conditions and determined that nighttime *g*_s_ was minimal at our site for both species (see Note S1 and Table S1). In addition, we examined the relationship between nighttime sap-flow measurements and nocturnal VPD (from 0300–0500 h), and concluded that nocturnal sap-flux for both species at our site was minimal (see Note S1 and Fig. S1).

A second source of error encountered in sap-flow methodology is a failure to assess the radial pattern of sap-flux (*J*_S_) with sapwood depth (see Phillips et al. 1996; Delzon et al. 2004; and Ford et al. 2004). In our study, we used sap-flow probes that were only 10 mm in length, and this allowed us to install probes in stems of variable diameter while largely avoiding the issue of probe insertion into stem heartwood (across piñon trees with sap-flow sensors, sapwood depth averaged 2.8 cm (±0.12), with a range of 1.1 to 5.2 cm). However, for some of the larger trees, this created a situation where we only measured a portion of the sapwood profile. To assess whether *J*_S_ decreased radially in larger stems, we constructed probes of varying length and installed sap-flow probes at depths of 0–10, 10–20, 20–30 mm in select large piñon (*n *=* *4) and juniper (*n *=* *3) trees. For the piñon trees used in this radial *J*_S_ analysis, sapwood depth averaged 2.9 cm, with a range of 2.4 to 3.3 cm depth. Due to the asymmetrical patterns of stem sapwood in juniper, we could not make accurate measurements of average sapwood depth in this species; however, we did take great care to insure that sap-flow probe installation into heartwood was avoided (i.e., we only installed radial sap-flow probes in zones of the stem where juniper sapwood depth was >20 mm). Over a 6-month period of measurements that spanned both dry and wet conditions, we did not detect a significant decline in overall mean radial sap-flow with depth for either species (see Fig. S2). Therefore, we concluded that our measurement of sap-flow at 0–10 mm sapwood depth was sufficient to accurately assess sap-flow rates for the two species monitored in our study.

Plant hydraulic conductance (*k*_s_, per unit sapwood area; in mol m^−2^ s^−1^ MPa^−1^) was calculated using the following relationship (1) based on Darcy's law (Wullschleger et al. 1998; Sperry et al. 2002) as follows:

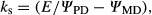
1with *E* equal to midday *J*_S_ per unit sapwood area (mol m^−2^ s^−1^) measured from 1100 to 1400 h and *Ψ*_PD_ and *Ψ*_MD_ representing soil (*Ψ*_S_) and midday leaf (*Ψ*_MD_) water potential (MPa), respectively. Due to the short stature of our monitored trees (mean height = 4.0 m, range 2.2 to 6.3 m), we did not account for any height/gravitational effects in our *k*_s_ assessments. For calculations of *k*_s_, only measurements with a water potential gradient of at least 0.5 MPa difference between *Ψ*_PD_ and *Ψ*_MD_ were retained in the analysis (hereafter, the midday water potential gradient is referred to as “Δ*Ψ*”). Values of Δ*Ψ* less than 0.5 MPa were excluded from *k*_s_ calculations because the assumption of predawn equilibrium of *Ψ*_PD_ and *Ψ*_S_ is invalid in isohydric piñon pine during extreme drought due to stomatal closure and hydraulic isolation from the soil (Plaut et al. 2012).

Additionally, in one of our treatment replicates (the flat aspect block), measurements of leaf stomatal conductance (*g*_s_) and net photosynthesis (*A*_n_) were made across the growing season in years 2010 and 2011 in both piñon and juniper. For a subset of monitored trees, leaf gas exchange was measured on the same dates when stem *J*_S_, and foliar *Ψ*_PD_ and *Ψ*_MD_ were measured in 2010 and 2011. This allowed us to directly assess the effect of reduced *k*_s_ on canopy carbon assimilation across both species at varying levels of water availability. Leaf gas exchange was measured using three portable gas exchange systems (LI-6400, Li-Cor Inc., Lincoln, NE). For details on the methodology, sampling periods, and results from the gas exchange sampling, see Limousin et al. (2013).

### Plot-level canopy transpiration and conductance

Woody canopy transpiration per unit ground area (*E*_C_, mm day^−1^) was calculated by summing individual transpiration estimates (mm day^−1^) for piñon and juniper in a particular plot. Species-specific estimates of daily transpiration per unit ground area (*E*_Ci_, mm day^−1^) were estimated according to the following expression (2):

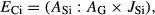
2with *A*_Si_ : *A*_G_ representing species-specific sapwood area per unit ground area (m^2^ m^−2^) and *J*_Si_ representing the daily cumulative sap-flux density (g** **m^−2^ day^−1^) for a given species (see Fig. S3 for annual sap-flow totals). The sap-flux density rate for a given species (by plot) was calculated by taking the average across all trees in which sap-flow was measured within a specific plot, and daily & annual estimates for *E*_C_ were subsequently calculated at the treatment level by averaging across all plots within a specific treatment class.

Canopy conductance per unit ground area (*G*_C_, mm s^−1^) was calculated at a daily time step (see Phillips and Oren 1998) as (3);

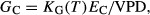
3where *K*_G_ is a conductance coefficient (115.8 + 0.4236*T*; kPa m^3^ kg^−1^) that incorporates temperature (*T*) effects on the psychrometric constant, latent heat of vaporization, specific heat of air, and density of air (Ewers and Oren 2000). Daily mean canopy *G*_C_ was calculated for the woody canopy using 24-h canopy transpiration (i.e., *E*_C_, mm day^−1^, normalized for daylight hours) and mean *T* and VPD averaged across daylight hours (i.e., PAR > 0). Daily *G*_C_ for days with daylight mean VPD < 0.1 kPa and daylight mean *T *<* *0°C was filtered from the data set.

Plot-level sapwood and canopy leaf area was estimated for piñon and juniper (Table1) using measurements of stem diameter and species-specific allometric equations that were developed on-site in 2007 (see Note S2 for model coefficients). Plot-level stem inventories that measured diameter and mortality status (alive or dead) of all woody stems were performed in 2007, 2011, and 2014. Whole-plot inventories along with monthly mortality surveys of designated sample trees were used in conjunction to estimate whole-plot mortality percentage trends from 2007 through 2012. Biomass values reported in Table1 and Fig. S4 reflect conditions present on the ground at the time treatments were initiated in 2007 (prior to mortality events). Finally, we assessed the appropriateness of using sapwood allometric models constructed in 2007 to predict sapwood in subsequent years following multiple years of whole-plot experimental treatments. In 2012, we measured sapwood area (via increment coring) in large branches and boles from a subset of piñon trees from irrigated, droughted, and ambient plots to verify that measured sapwood area was consistent with predictions made using the 2007 piñon allometric model (see Fig. S5). The same verification using coring measurements was not applicable for juniper due to the highly asymmetrical distribution of juniper sapwood.

**Table 1:** Descriptive statistics for *Pinus edulis* and *Juniperus monosperma* trees across all plots and experimental treatments in year 2007 (baseline) and 2012 (with mortality effects)

Biomass Parameter	Irrigation year-2007 (*n *=* *3)	Ambient year-2007 (*n *=* *3)	Drought year-2007 (*n *=* *3)	Irrigation year-2012 (*n *=* *3)	Ambient year-2012 (*n *=* *3)	Drought year-2012 (*n *=* *3)
Total basal area (m^2^ ha^−1^)	24.3 (2.1)	20.0 (1.7)	17.6 (0.2)	23.8 (2.0)	19.8 (1.7)	12.2 (2.6)
Piñon basal area (m^2^ ha^−1^)	5.2 (0.8)	2.4 (0.3)	1.9 (0.4)	4.7 (0.7)	2.2 (0.4)	0.6 (0.2)
Juniper basal area (m^2^ ha^−1^)	19.1 (1.8)	17.7 (1.4)	15.7 (0.6)	19.1 (1.8)	17.6 (1.4)	11.6 (2.6)
Total sapwood area (m^2^ ha^−1^)	4.83 (0.46)	3.21 (0.25)	2.82 (0.28)	4.65 (0.39)	3.13 (0.32)	1.68 (0.23)
Piñon sapwood area (m^2^ ha^−1^)	2.30 (0.36)	1.04 (0.11)	0.87 (0.18)	2.12 (0.27)	0.97 (0.16)	0.28 (0.10)
Juniper sapwood area (m^2^ ha^−1^)	2.53 (0.21)	2.17 (0.14)	1.95 (0.11)	2.53 (0.21)	2.16 (0.15)	1.40 (0.16)
Total stem density (>5 cm, ha^−1^)	567 (46)	350 (22)	354 (85)	n/a	n/a	n/a
Piñon stem density (>5 cm, ha^−1^)	173 (20)	77 (15)	69 (13)	n/a	n/a	n/a
Juniper stem density (>5 cm, ha^−1^)	394 (30)	273 (35)	285 (74)	n/a	n/a	n/a
Leaf area index (m^2^ m^−2^)	1.09 (0.10)	0.77 (0.06)	0.68 (0.04)	n/a	n/a	n/a
Woody Canopy Coverage (%)	47.5 (1.9)	36.6 (3.0)	32.3 (4.1)	n/a	n/a	n/a

Basal area estimates are based on diameter measured at 30-cm stem height. Sapwood area and LAI were predicted using allometric equations developed on-site in 2007. For individuals with multiple stems at 30-cm height, a single equivalent diameter was calculated. Woody canopy coverage was estimated from aerial photographs of the study site and included all woody shrubs and trees. Annual mortality estimates for each species (by plot) were developed from periodic tree/canopy dieback surveys performed on target trees in each plot and whole-plot stem inventories and mortality status checks performed in 2007, 2011, and 2014. Error bars represent (±1 SE) of the mean.

### Statistical analysis

Where appropriate, statistical tests for significant treatment differences across the 5+ year measurement period (regardless of variable) were performed using linear mixed-effects models with a repeated measures and random effects covariance structure. All analyses were performed using SAS software and the PROC MIXED procedure (SAS v9.3; SAS Institute, Cary, NC). Significance was evaluated at the *α *= 0.05 threshold level unless otherwise stated in the text. Where appropriate, final model selection was based on Akaike information criterion (AIC) and reduction of model root mean square error (RMSE). For the repeated measures analysis, we used an autoregressive first-order [AR(1)] covariance structure (with day as the repeated factor, and tree ID as the subject). In all statistical tests, year and replicate block were treated as random effects. The inclusion of a repeated measures covariance structure was critical as it significantly improved model AIC, reduced model RMSE, and controlled for the number of degrees of freedom used in the mixed-model F-tests.

To assess the response of physiological parameters to variations in soil water availability, atmospheric evaporative demand, and light, we used ANCOVA models (via PROC MIXED) to analyze the response of midday *J*_S_, *k*_s_, and Δ*Ψ* to variations in *Ψ*_PD_, VPD, and PAR. With the exception of climate variables, all mean and SE values (±1 SE) reported in the text for plant physiological parameters are based on estimates obtained via the LSMEANS procedure in the PROC MIXED model analyses. To assure that the model assumptions of equal variance and normal distribution of residuals were achieved (including linearity assumptions for ANCOVA), we performed all statistical tests using both nontransformed and transformed response variables (via sqrt. and log transformations). In all cases, the statistical inference of significance (at *α *= 0.05) for treatment effects, contrast tests, and parameter estimates did not change between models utilizing nontransformed and transformed response variables. Therefore, we present and report the statistical results for the nontransformed model results in the Results and Discussion. For an example of the similar statistical inferences based on nontransformed and transformed response variables, please see ANCOVA model results (Tables S2 and S3).

## Results

### Climate and treatment effects on plant water stress

**Figure 1:**
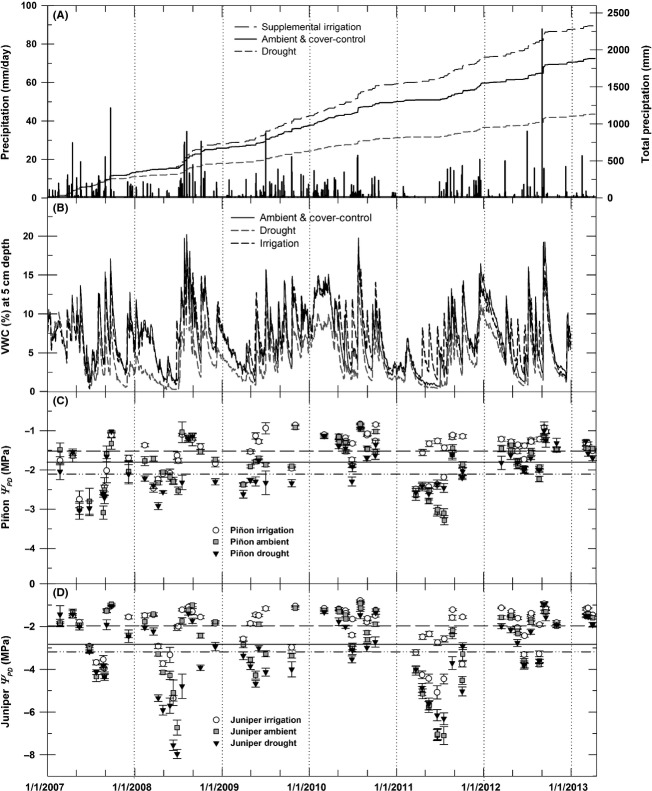
Climate summary for 5+ year period starting in 2007. (A) Daily ambient precipitation and total precipitation by treatment across 5+ year period, (B) VWC at 5-cm soil depth by treatment, and predawn water potential for piñon (C) and juniper (D) by treatment (*Ψ*_PD_ values are mean ± 1 SE). For panels 1C and 1D, horizontal lines indicate 5+ year treatment mean for each species as follows: irrigation (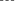), ambient (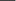), and drought (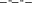).

During the 5 years of this study, ambient precipitation was highly variable, ranging from an annual low of 252.0 mm year^−1^ in 2011 up to 341.3 mm year^−1^ in 2007 (Fig.1A). Mean annual precipitation during 2007–2012 was 305.7 (±1 SE = 13.7) mm year^−1^, which was 85% of the 20-year mean for the site (362.7 mm year^−1^, 1989–2009). A total of 442.5 mm of water were added to irrigation plots during 2007–2012, increasing total irrigation precipitation to 23.5% above ambient (Fig.1A). Drought plot precipitation (Fig.1A) averaged 164.2 (±7.9) mm year^−1^ (45% deduction from ambient, starting summer 2007). Due to low atmospheric humidity and relatively few periods with prolonged cloud cover, evapotranspiration at our site is rarely limited by a lack of available energy or low daytime temperatures during the growing season. This is evidenced by sustained periods with high daily maximum VPD, high daily temperatures, and high midday PAR at our site throughout the growing season (defined as March through October for this analysis, Fig. S6).

Soil water content (VWC at 5 cm depth) and plant water potential (*Ψ*_PD_) varied on an annual, seasonal, and treatment basis across the 2007–2012 period (Fig.1, panels B–D). Compared to ambient plots, the drought treatment infrastructure consistently lowered soil VWC, especially during periods of regular precipitation pulses and higher ambient VWC. Irrigation treatments significantly increased VWC following irrigation events, which is clearly evident during prolonged drought periods such as the spring and early summer of 2011 (Fig.1B). A more detailed analysis and discussion of treatment effects on both shallow and deep soil VWC has been presented elsewhere (see Pangle et al. 2012 and Plaut et al. 2013). For the purpose of assessing plant response to soil water stress, this analysis utilized predawn leaf water potential (*Ψ*_PD_), which is a useful metric to assess plant water stress as it integrates the ability of plants to access and uptake water regardless of variation in VWC with soil depth.

Across the 5+ year sampling period (Fig.1C–D), drought treatment infrastructure significantly reduced *Ψ*_PD_ (for both species) compared to ambient (*P < *0.02), while irrigated trees (both species) exhibited higher mean *Ψ*_PD_ compared to ambient trees (*P *<* *0.0001). Spanning the range of water availability imposed by our treatments, *Ψ*_PD_ in irrigated, ambient, and droughted piñon (Fig.1C) averaged −1.51 (±0.04), −1.81 (±0.04), and −2.10 (±0.06) MPa, respectively, across the 5+ year period. In contrast, *Ψ*_PD_ in juniper (Fig.1D) averaged −1.96 (±0.12), −2.80 (±0.12), and −3.18 (±0.11) MPa in the irrigated, ambient, and drought treatments, respectively.

For both species, there were no significant differences in *Ψ*_PD_ between ambient and cover-control trees (*P *=* *0.82 for piñon, *P *=* *0.64 for juniper, data not shown). Midday leaf water potential (*Ψ*_MD_) also differed across treatments in both species (*P *<* *0.05, irrigation and drought contrasted with ambient) with irrigated, ambient, and droughted *Ψ*_MD_ averaging −2.29 (±0.03), −2.38 (±0.03), and −2.51 (±0.04) MPa for piñon and −2.88 (±0.09), −3.52 (±0.10), and −3.81 (±0.09) MPa for juniper, respectively, across the 5+ year period (see Fig. S7). There were no significant differences in *Ψ*_MD_ between ambient and cover-control trees for either species (*P *=* *0.35 for piñon, *P *=* *0.56 for juniper, data not shown).

**Figure 2:**
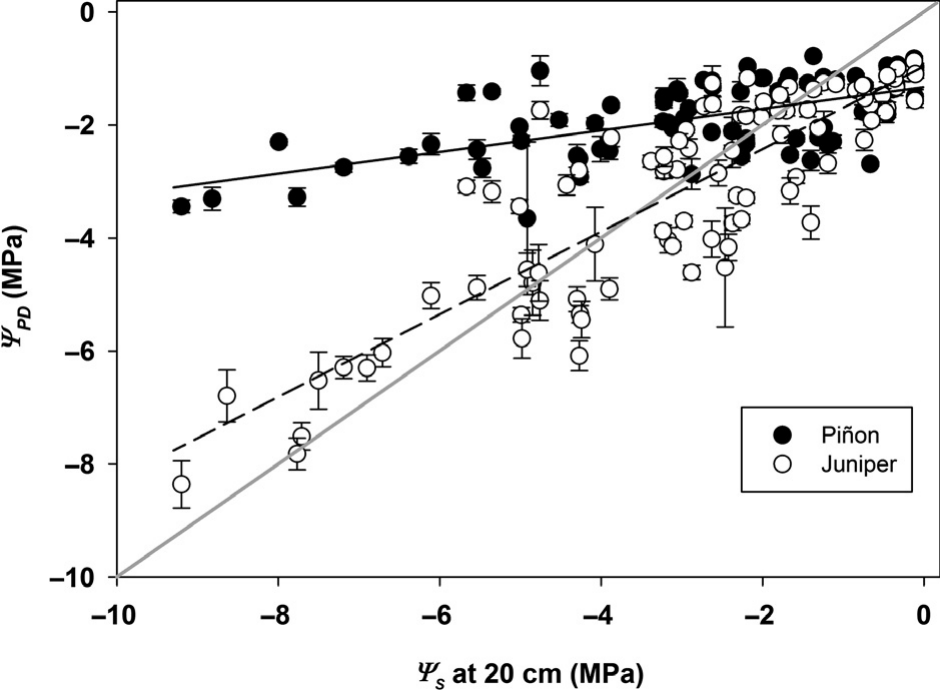
Relationship between foliar predawn water potential (*Ψ*_PD_) and soil water potential (*Ψ*_s_) at 20 cm depth (measured via soil psychrometers) for piñon and juniper across a 5+ year period from 2007 through 2012. Error bars represent ±1 SE of the mean. For each species, data were pooled and a single regression relationship was fit across treatments.

During the driest years (2008 and 2011), mean juniper *Ψ*_PD_ reached lows of between −6 and −8 MPa in both ambient and drought treatments (Fig.1D). Juniper at our site exhibited anisohydric behavior, maintaining hydraulic connections with the soil and tracking declines in soil water availability (Fig.2), while also maintaining low, but significant canopy gas exchange even as *Ψ*_s_ and *Ψ*_PD_ declined down to values of −6 to −7 MPa during drought (Limousin et al. 2013). Predawn water potential (*Ψ*_PD_) in piñon deviated significantly from soil water potential (*Ψ*_s_) during periods of drought (Fig.2). Piñon mean *Ψ*_PD_ did not decline below −3.5 MPa during the driest conditions, periods when both juniper foliar *Ψ*_PD_ and soil *Ψ*_s_ (*Ψ*_s_ from soil psychrometer measurements) declined to −8 to −10 MPa (Fig.2). Relatively isohydric piñon was hydraulically disconnected from the surrounding soil drought, closing stomata to prevent further canopy water loss once overall *Ψ*_s_ within the rooting zone declined below a threshold of −2.5 to −3.0 MPa, thus limiting the development of excessive xylem tensions within the aboveground water conducting tissue.

### Transpiration and plant hydraulic conductance – treatment effects

**Figure 3:**
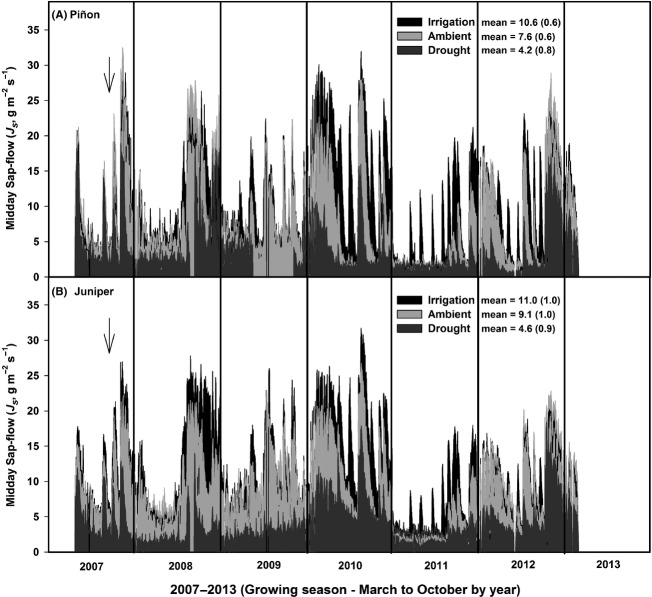
Midday sap-flow patterns (*J*_S_) for irrigation, ambient, and drought treatments across the 5+ year study period (panel A – piñon, panel B – juniper). For each year, data are the daily mean midday *J*_S_ rate (1100 to 1400 h) across all days from March to October. The 5+ year treatment mean for each species is provided in the figure legends. Drought treatments were fully implemented on day 239, year 2007 (downward arrow). Error bars were not included to preserve clarity and viewability of the plots. Midday *J*_S_ error estimates for treatment means are provided in Fig. S10 regression plots.

Our precipitation manipulation resulted in a wide range of transpiration responses in both species (see Fig.3, for March to October sap-flow patterns by year). Sap-flow (*J*_S_, midday hours, g m^−2^ s^−1^) was significantly reduced (*P *<* *0.01) in both droughted piñon and juniper during the 2007–2012 period compared to ambient trees (Fig.3). Supplemental irrigation increased midday *J*_S_ following irrigation events for both species (especially during the dry year 2011). Mean *J*_S_ rates in irrigated piñon were significantly higher compared to ambient (*P *<* *0.001), but *J*_S_ rates for irrigated juniper did not differ significantly (*P *=* *0.18) from ambient trees overall. Across the 5+ year analysis period, midday *J*_S_ in irrigated, ambient, and drought plots averaged 10.6 (±0.58), 7.6 (±0.62), and 4.2 (±0.77) g m^−2^ s^−1^ for piñon and 11.0 (±1.01), 9.1 (±1.02), and 4.6 (±0.85) g m^−2^ s^−1^ for juniper (Fig.3). There were no significant differences in sap-flow between ambient and cover-control trees (see Fig. S8) for either species over the 5+ year period (*P *=* *0.98 for piñon, *P *=* *0.78 for juniper).

**Figure 4:**
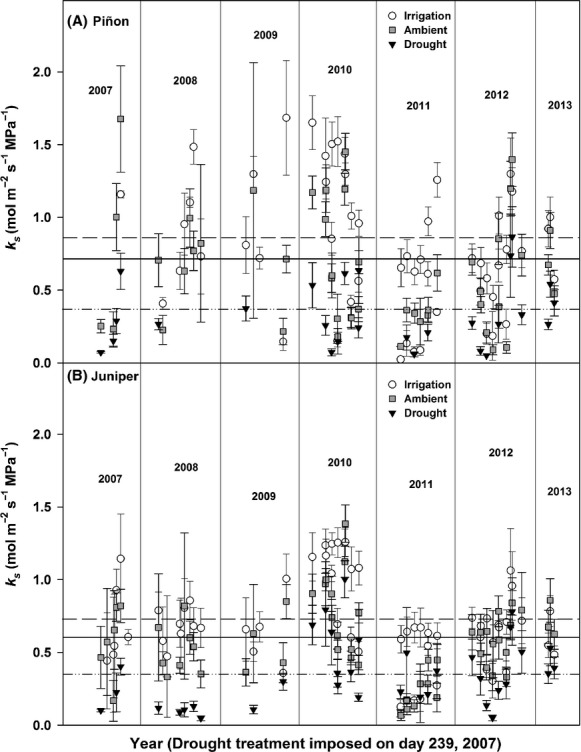
Plant hydraulic conductance patterns (*k*_s_) for irrigation, ambient, and drought treatments across the 5+ year study period (panel A – piñon, panel B – juniper). Horizontal lines indicate 5+ year treatment mean for each species as follows: irrigation (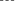), ambient (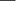), and drought (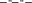). Error bars represent ±1 SE of the mean.

Experimentally imposed drought reduced plant hydraulic conductance (*k*_s_) in both piñon and juniper compared to *k*_s_ in ambient trees (*P *<* *0.01; Fig.4), while water additions significantly increased *k*_s_ (*P *<* *0.05) compared to ambient piñon. While mean *k*_s_ in irrigated juniper was higher than ambient trees, the increase was not significant (*P *=* *0.17) overall. Across this analysis period, *k*_s_ in irrigated, ambient, and drought plots averaged 0.864 (±0.052), 0.711 (±0.053), and 0.368 (±0.077) mol m^−2^ s^−1 ^MPa^−1^ for piñon and 0.725 (±0.062), 0.602 (±0.063), and 0.353 (±0.054) mol m^−2^ s^−1 ^MPa^−1^ for juniper (Fig.4). There were no significant differences in *k*_s_ between ambient and cover-control trees (data not shown) for either species over the 5+ year period (*P *=* *0.44 for piñon, *P *=* *0.32 for juniper).

### Response of *k*_s_ and *E* to *Ψ*_PD_, VPD, and PAR

**Figure 5:**
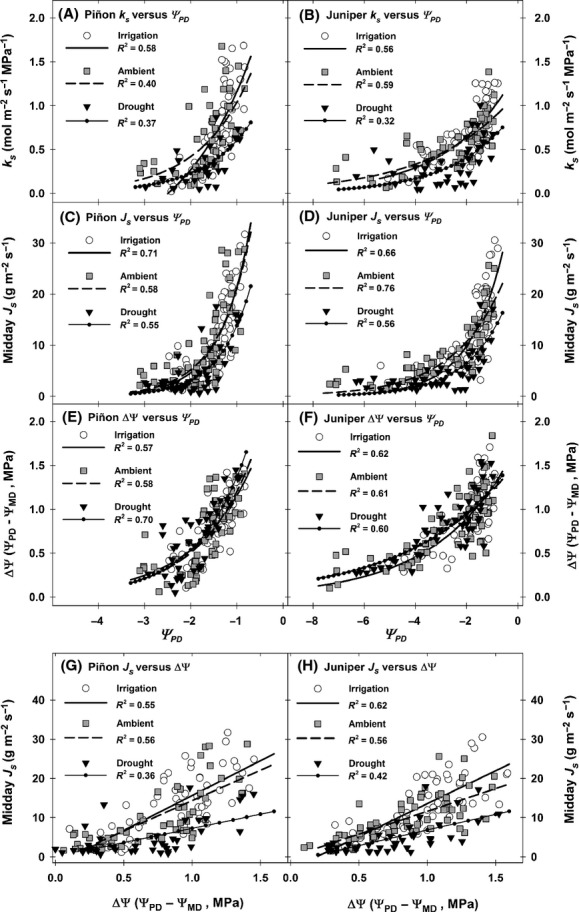
Relationship between *k*_s_ and predawn water potential (*Ψ*_PD_) across the 5+ year study period for each species and treatment (panels A and B). Relationship between midday sap-flow (*J*_S_) and *Ψ*_PD_ across the study period (panels C and D). Response of Δ*Ψ* (*Ψ*_PD _− *Ψ*_MD_) to plant water stress (*Ψ*_PD_) across the study period (panels E and F). Response of midday *J*_S_ to Δ*Ψ* across the study period (panels G and H). For panels A–F, nonlinear regression fits are the best-fit relationship using a two-parameter exponential function of the form; *y = ae*^*bx*^. Error bars were not included to preserve clarity and viewability of the plots (note: error bars for treatment means are provided in Figs S9 and S10).

We examined the response of both *k*_s_ and midday *J*_S_ (i.e., *E*) to *Ψ*_PD_, midday VPD, and midday PAR across the 5+ year period. In mixed-model analyses (by species) regressing either *k*_s_ or midday *J*_S_ against the independent variables *Ψ*_PD_, VPD, and PAR, we found that *Ψ*_PD_ and midday VPD significantly influenced *k*_s_ and midday *J*_S_ responses (*P *<* *0.05), but midday PAR did not (*P *>* *0.10). Therefore, we dropped PAR from the analysis and tested reduced ANCOVA models regressing either *Ψ*_PD_ or midday VPD against the response variables *k*_s_ and midday *J*_S_. In all cases, the reduced ANCOVA models utilizing only *Ψ*_PD_ had better predictive power and lower (i.e., better) Akaike information criterion (AIC) fit statistics compared to ANCOVA models that only utilized VPD as the predictor variable. This demonstrates that our semiarid site is much more water limited than energy constrained in regard to realized evapotranspiration. Thus, while VPD was a significant predictor of both midday *J*_S_ and *k*_s_ response in many models, it did not have the predictive power of *Ψ*_PD_, so we restricted the majority of our ANCOVA analyses to models that incorporated *Ψ*_PD_ as the sole predictor (Fig.5A–F).

### Response of *k*_s_ and *E* to drought

The main limit to transpiration during warmer months at our site is soil water availability, and accordingly both *k*_s_ and midday *J*_S_ (i.e., *E*) exhibited a significant and positive relationship with *Ψ*_PD_ regardless of treatment or species (Fig.5A–D). This relationship is particularly clear when comparing years with contrasting water availability. During the drought conditions of 2011, both *k*_s_ and midday *J*_S_ showed a sharp reduction compared to 2010 – a year when soil moisture was more abundant during the winter, spring, and monsoon periods (Figs1A, 3, and 4).

For piñon and juniper, when *k*_s_ and midday *J*_S_ were regressed against *Ψ*_PD_ (Fig.5A–D) there were significant differences among the intercepts by treatment (ANCOVA, Tables S2 and S3), indicating that the treatments resulted in a significant modification of plant hydraulic function under the majority of nonlimiting water conditions observed. Across all species and treatments, Δ*Ψ* decreased (Fig.5E,F) as *Ψ*_PD_ decreased during drought (both ambient and experimentally imposed). Interestingly, while mean Δ*Ψ* values varied among treatments along with *Ψ*_PD_, the maximum observed value for Δ*Ψ* within species did not differ across treatments (Fig.5E,F), with maximum Δ*Ψ* values of ∼1.5 MPa observed across all piñon treatments and a maximum Δ*Ψ* of ∼1.5 to 1.8 MPa observed across treatments for juniper (see intercept estimates, Tables S2 and S3). We observed a significant relationship between sap-flow and Δ*Ψ* across species and treatments (Fig.5G,H). Compared to ambient trees, droughted trees of both species exhibited a significantly weaker *J*_S_ response as *ΔΨ* increased. Notably, this decreased *J*_S_ response to Δ*Ψ* is a clear indication of prolonged and persistent decreases in *k*_s_ for droughted trees, even under conditions with more favorable *Ψ*_s_ (Fig.5E–H, Tables S2 and S3). For isohydric species such as piñon, which require foliar abscisic acid (ABA) signaling to fully maintain stomatal closure during drought, the persistence of lower *k*_s_ at higher *Ψ*_s_ indicates that reduced hydraulic function persisted even after stomatal inhibition, due to high levels of foliar ABA, diminished (Brodribb and McAdam 2013).

### Effects of prolonged reductions in *k*_s_

**Figure 6:**
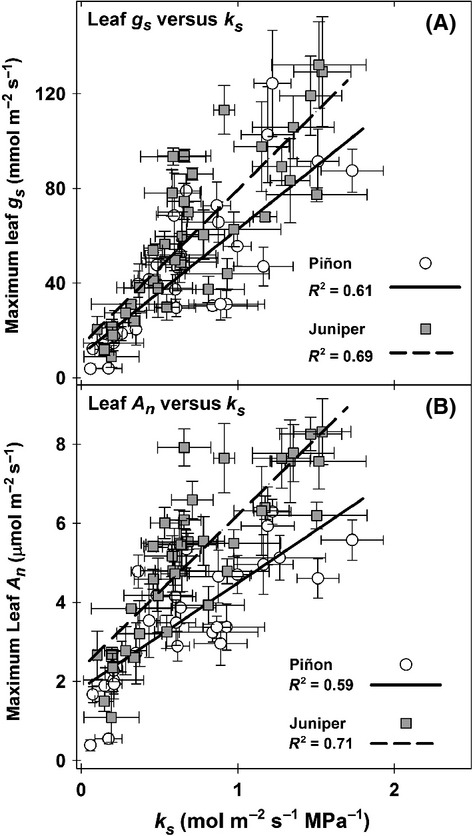
Relationship between maximum stomatal conductance (*g*_s_) and whole-plant *k*_s_ during years 2010 and 2011 by species (panel A). Relationship between maximum net photosynthesis (*A*_n_) and whole-plant *k*_s_ during years 2010 and 2011 by species (panel B). Data points are treatment means on a particular measurement date (±1 SE). All data represent dates when concurrent data from both leaf gas exchange measurements and stem sap-flow measurements (for *k*_s_) were available. For each species, a single regression relationship was fit across treatments (see Limousin et al. 2013 for details on the gas exchange sampling effort).

In addition to assessments of canopy *E* and whole-plant conductance (*k*_s_) using sap-flow measurements, we also compared our sap-flow estimates of *k*_s_ to leaf-level gas exchange rates measured in years 2010 and 2011 (Fig.6A,B). For both species, stomatal conductance (*g*_s_) and net photosynthesis (*A*_n_) exhibited a strong and significant decline at lower *k*_s_ values. Thus, chronically low *k*_s_ corresponded to low canopy gas exchange and net carbon assimilation.

**Figure 7:**
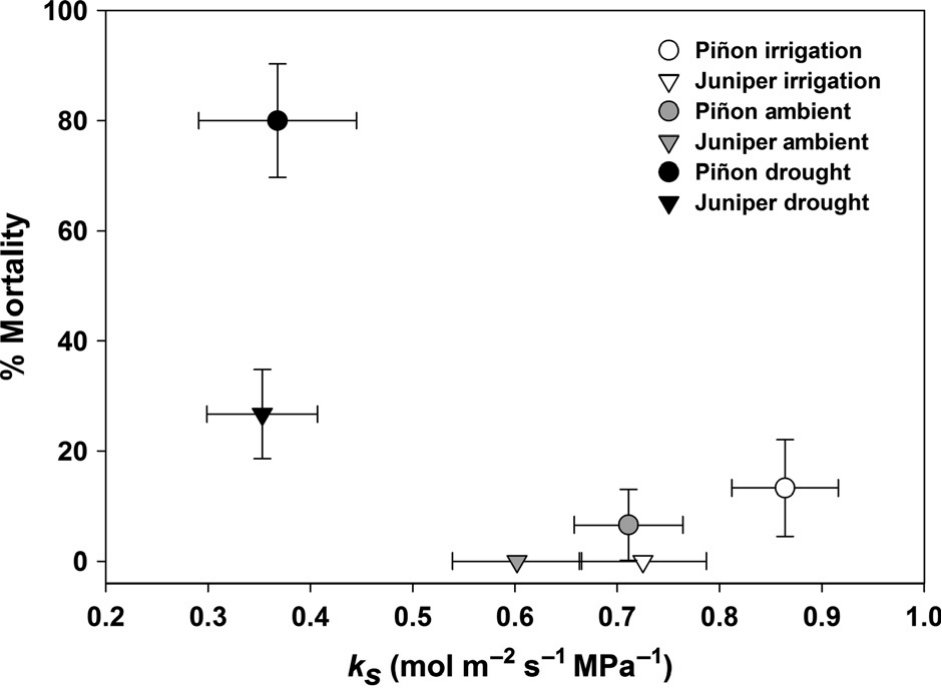
Relationship between mortality rate (%) and plant hydraulic conductance (*k*_s_) across the 2007 through 2012 study period. Error bars represent ±1 SE. For the mortality percentage estimates, the reported mean represents the average across plots within a given treatment (i.e., individual mortality percentage estimates were calculated for each plot by species, then averaged across plots, with the associated error estimates being calculated using a “standard error estimation for proportions” procedure).

Over the 5+ year time frame of this analysis, experimentally droughted and intensively monitored piñon and juniper spent more time at low *k*_s_ levels compared to either ambient or irrigated trees (Fig.7). Concurrently over this time frame, we observed significant mortality and canopy dieback for intensively monitored trees in our experimentally droughted plots, with droughted piñon trees experiencing 80% mortality through year 2012 (Fig.7) and droughted juniper trees experiencing 27% mortality and/or overwhelming canopy dieback (≥85% canopy loss) through year 2012 (Fig.7).

Piñon mortality in droughted plots was initially observed in year 2008, and by year 2012 piñon basal and sapwood area across all droughted plots was reduced to 0.60 (±0.20) m^2^ ha^−1^ and 0.28 (±0.10) m^2^ ha^−1^, respectively (Table1). In contrast, juniper mortality and/or canopy dieback (initially observed in 2009) reduced total juniper basal and sapwood area in drought plots to 11.6 (±2.6) m^2^ ha^−1^ and 1.40 (±0.16) m^2 ^ha^−1^ by year 2012 (Table1).

### Daily canopy *E*_C_ and *G*_C_

**Figure 8:**
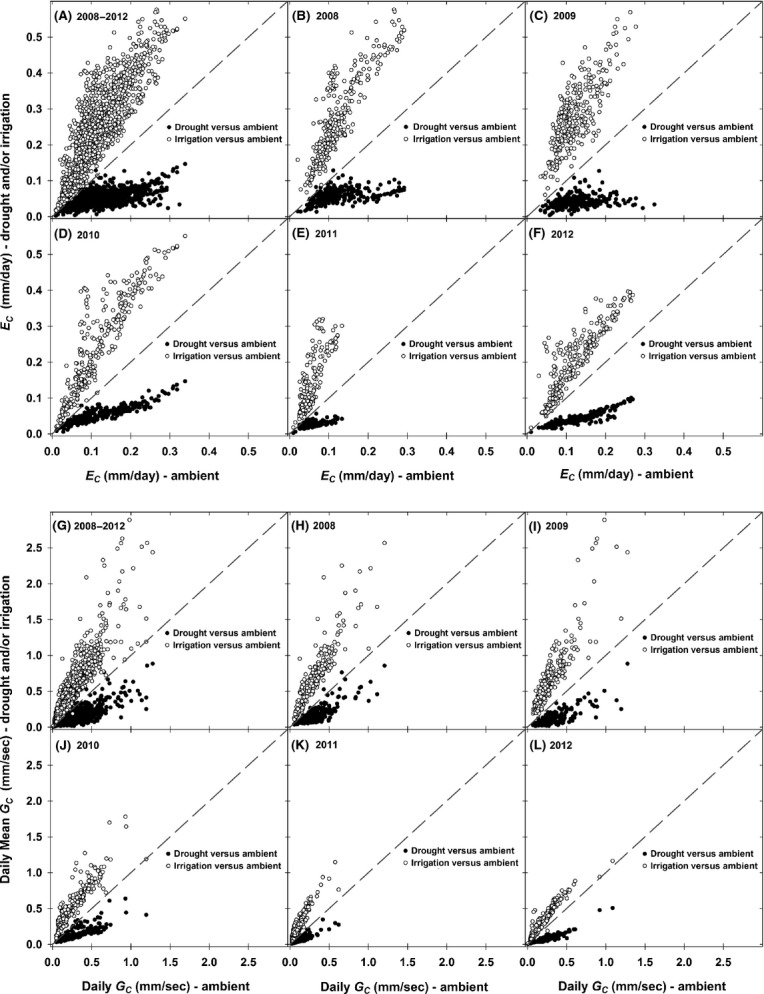
Daily canopy transpiration (*E*_C_, mm day^−1^) and woody canopy conductance (*G*_C_, mm s^−1^) across the 2008–2012 period (panels A and G) and by year (panels B–F and H–L). The relationship between daily total *E*_C_ and *G*_C_ in irrigated plots versus ambient plots is plotted in open symbols, and the relationship between droughted plots and ambient is plotted in closed symbols. The dashed line represents the 1:1 relationship between ambient plots (*x*-axis) and either irrigated and/or droughted plots (*y*-axis). Daily mean canopy *G*_C_ is the average value across daylight hours calculated using total 24-h transpiration (i.e., *E*_C_, mm day^−1^, normalized for daylight hours) and mean temperature and VPD across daylight hours (PAR > 0). Daily G_C_ for days with daylight mean VPD < 0.1 kPa has been filtered from the analysis. Error bars (variance between plots within a treatment) have been omitted to preserve clarity.

Daily woody canopy transpiration (*E*_C_) in ambient plots reached a maximum value of 0.34 (±0.05) mm day^−1^ across the 2008–2012 period (Fig.8, panels A–F). In contrast, daily *E*_C_ in droughted plots reached maximum rates of only 0.15 (±0.04) mm day^−1^ during the same period (Fig.8, panels A–F). Daily *E*_C_ in irrigated plots ranged up to 0.58 (±0.02) mm day^−1^, due partly to higher stem densities for both piñon and juniper, and hence higher total sapwood area in irrigated plots (Fig.8, Table1). It should be noted that higher stem density in irrigated plots was not a treatment effect, as these conditions were present at the time of treatment initiation in year 2007 (see Table1). Maximum daily *E*_C_ values observed across ambient plots during the 2008–2012 period (i.e., 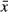 = 0.339 mm day^−1^, ±0.046) were almost identical to maximum *E*_C_ rates (0.34 mm day^−1^) reported by West et al. (2008) in a piñon–juniper woodland in southern Utah with comparable basal area (18.4 m^2^ ha^−1^) and species composition, while maximum daily *E*_C_ values observed in irrigation plots (0.58 mm day^−1^, ±0.02) were also in the range (0.19–0.99 mm day^−1^) of mean daily *E*_C_ rates observed and/or modeled in various other pinon and juniper woodlands (Lane and Barnes 1987; Leffler et al. 2002).

**Figure 9:**
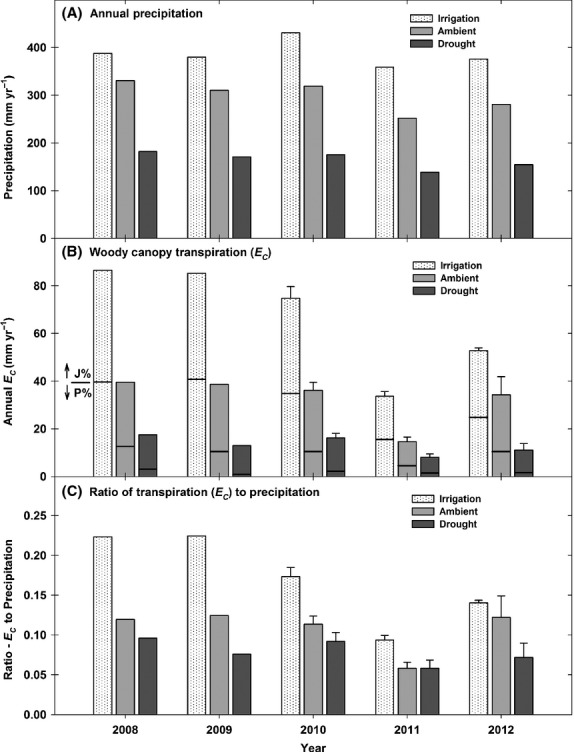
Annual precipitation (panel A), woody canopy transpiration (panel B), and the ratio of total transpiration to precipitation (panel C) across the 2008–2012 period by treatment. Whole-plot transpiration estimates were based on measurements from one replicate block (*n *=* *1) in years 2008 and 2009, and all replicate blocks (*n *=* *3) in years 2010 through 2012. For years 2010–2012, error bars (±1 SE, panels B and C) represent the variation between plots (by treatment). The proportion of total annual *E*_C_ attributable to either juniper (J%) or piñon (P%) transpiration is designated by the black horizontal line located within in each bar graph in panel B.

Across the 2008–2012 period at our site, daily mean woody canopy *G*_C_ averaged 0.25 (±0.04), 0.11 (±0.02), and 0.51 (±0.05) mm s^−1^ for ambient, drought, and irrigation plots, respectively (Fig.8, panels G–L), with maximum daily mean *G*_C_ values up to 1.27, 0.88, and 2.88 mm s^−1^ observed in ambient, drought, and irrigated plots, respectively, in year 2009 (Fig.8, panel I). Annual *E*_C_ totals across treatments ranged up to 39.5, 17.5, and 86.5 mm year^−1^ for ambient, drought, and irrigation plots, respectively, across the 2008–2012 period (Fig.9, panel B). Daily *E*_C_, Daily *G*_C_, and annual *E*_C_ were sharply reduced due to naturally occurring drought in year 2011 (Figs8 and 9), a year when ambient precipitation totaled 252.0 mm year^−1^ and drought treatment plots only received 138.6 mm year^−1^ (Fig.9, panel A). The percentage of total *E*_C_ represented by piñon transpiration was 30%, 17%, and 47% of total annual *E*_C_ in ambient, drought, and irrigated plots, respectively, across the 2008–2012 period (Fig.9, panel B). The low contribution of piñon transpiration to total annual *E*_C_ in droughted plots was due to the high level of piñon mortality in droughted plots and the associated strong reductions in total piñon sapwood area (Fig.7, Table1). After accounting for piñon *E*_C_, the remainder of total annual *E*_C_ (regardless of treatment) was due solely to juniper transpiration (Fig.9, panel B), as the woody canopy at our site was almost exclusively composed of piñon and juniper stems. Notably, the percentage of total annual precipitation transpired by the piñon–juniper canopy was quite low, averaging just 11, 8, and 17% across the 2008–2012 period in ambient, drought, and irrigated plots, respectively (Fig.9). This low ratio of total annual *E*_C_ to precipitation is consistent with observations from other studies in piñon–juniper woodlands. Specifically, West et al. (2008) observed annual *E*_C_ to precipitation ratios in a piñon–juniper woodland in southern Utah that ranged from 6 to 14% across years that varied in total precipitation from approximately 150 to 340 mm year^−1^.

## Discussion

### Long-term precipitation reduction, induced *k*_s_ reductions, and mortality likelihood

The substantial long-term decreases that we observed in hydraulic conductance (*k*_s_) of both species under imposed drought (41 and 48%, juniper and piñon, respectively), and the corresponding increases in *k*_s_ with supplemental irrigation (20 and 22%, respectively) reflect the functional consequences of sustained manipulation of soil water availability (Fig.4). Our assessment of changes in whole-plant *k*_s_ allowed for an examination of how changes in the capacity of the xylem (and rhizosphere/soil flow path) to supply water ultimately affected both the physiological consequences of severe drought and the survival of the individual. Reduced *k*_s_ had a strong impact on leaf-level stomatal conductance and photosynthesis (Fig.6), and low *k*_s_ impacted mortality as predicted, with dying trees experiencing more severe reductions in *k*_s_ than those that survived (Fig.7). The consistency of this result suggests that both species experience hydraulic impairment and dysfunction prior to mortality, despite their different hydraulic and stomatal response strategies. Globally, decreased xylem- and/or leaf-specific hydraulic conductance has been directly implicated as one of the main factors associated with mortality, and this pattern has been observed for a number of conifer and angiosperm species (Martínez-Vilalta and Piñol 2002; Anderegg and Anderegg 2013; Anderegg et al. 2013, 2014; Barigah et al. 2013; Hartmann et al. 2013a; Mitchell et al. 2013; Sevanto et al. 2014). In addition, even in the absence of whole-tree mortality, significant branch and canopy dieback due to hydraulic dysfunction has been observed in various species subjected to either experimental or naturally occurring drought (Davis et al. 2002; Vilagrosa et al. 2003; Kukowski et al. 2013; Nardini et al. 2013).

### Precipitation pulses and reduced *k*_s_

It is well established that stomatal regulation of gas exchange during drought reduces carbon gain (Irvine et al. 1998; Hubbard et al. 2001; Vilagrosa et al. 2003). Moreover, the decreased *k*_s_ observed in droughted trees has important consequences for productivity and survival, especially in semiarid dryland systems where precipitation pulses are dynamic in nature (Loik et al. 2004; Schwinning and Sala 2004; Plaut et al. 2013). While we observed a resumption of *E* (via sap-flow) in surviving trees following smaller to medium size precipitation events (<50 mm), the responses were variable and related to treatment differences in *k*_s_ (Figs1, 3, and 4). In this same system, Plaut et al. (2013) observed significantly lower *E* responses to precipitation pulses (for events up to 20 mm) in droughted piñon and juniper trees that died, and they speculated that one of the mechanisms responsible for this pattern was a potential reduction in plant hydraulic conductance in droughted trees. Our results confirm this hypothesis. Specifically, trees with lower *k*_s_ must exhibit greater reductions in stomatal conductance under high VPD compared to nonstressed trees to prevent excessive declines in foliar *Ψ* (Sperry 2000; Franks 2004; Brodribb and Cochard 2009). Thus, a decline in *k*_s_ has several implications for drought stressed trees; severely droughted trees not only have much lower maximum *E* and canopy gas exchange rates compared to ambient trees (Figs3, 5, and 6), but also often have a reduced ability to return to predrought physiological function even after drought conditions have ended. Unlike trees in the ambient treatment, trees in the drought plots with significant reductions in *k*_s_ were unable to take full advantage of transient periods with higher VWC. This is certainly a short-term disadvantage in a semiarid system where trees rely on short-term pulses in soil VWC (see Plaut et al. 2013; Fig.1 inset). Incomplete recovery of canopy gas exchange following drought has been observed in both conifer and angiosperm species, and this lack of postdrought recovery has been directly linked with the degree of hydraulic impairment of the xylem during the preceding drought (Blackman et al. 2009; Brodribb and Cochard 2009; Resco et al. 2009; Poyatos et al. 2013; Urli et al. 2013; Anderegg et al. 2014).

### Implications and consequences of prolonged reductions in *k*_s_

Reduced *k*_s_ is not only a measure of the impairment and loss of hydraulic function but also has negative implications for the long-term carbon status of droughted trees (Bréda et al. 2006; McDowell 2011), as these trees will have decreased levels of photosynthesis and net carbon assimilation (Fig.6) both during and potentially following drought events (especially if significant time lags occur between drought cessation and full hydraulic recovery). The implications of decreased carbon assimilation and potential reductions in total nonstructural carbohydrate (NSC) levels extend beyond carbon demands imposed by respiratory requirements. Reduced NSC concentrations in droughted trees may limit the potential for osmotic adjustments to maintain turgor under drought conditions, limit the carbon substrates potentially needed for refilling and cavitation repair to occur, and limit sugars and starches needed for new root or xylem growth once drought is alleviated (McDowell 2011; McDowell et al. 2013). Reduced hydraulic function (low *k*_s_) and low water availability may also constrain phloem transport due to a lack of adequate water to maintain phloem functionality and translocation, and this can impact the ability of droughted trees to access and transport sugars, even if significant NSC reserves are present (Hartmann et al. 2013a,b; Sevanto et al. 2014). Furthermore, NSC reductions and hydraulic dysfunction may negatively impact the defensive capacity of droughted trees to successfully resist attack by biotic agents such as bark beetles (Bentz et al. 2010; McDowell 2011; Gaylord et al. 2013). Recent work by Dickman et al. (2014) in this same system demonstrated that declines in foliar starch were observed in both piñon and juniper prior to mortality, thus providing evidence that significant reductions in *k*_s,_ and subsequent declines in carbon fixation had a direct effect on total foliar NSC and starch concentrations and the survival time of droughted trees.

The broader consequences of prolonged reductions in whole-plant *k*_s_ extend beyond the individual to entire ecosystems and landscapes that are negatively impacted by drought-related mortality events. Our study clearly shows how strong reductions in plot-level *E*_C_ and *G*_C_ (and ultimately woody plant net carbon fixation) result from a combination of experimentally induced drought, reduced hydraulic function (lower *k*_s_), and subsequent stem mortality and reductions in woody canopy cover. This is especially apparent when the contribution of piñon canopy cover to total woody canopy cover is examined from a structural and physiological function perspective. Structurally, piñon in our study only comprised between 11 and 21% of total basal area (depending on treatment) in year 2007 (Table1). However, from a physiological perspective, the amount of totally active sapwood that was present in the piñon canopy cover in 2007 ranged from 31% in drought plots up to 48% in irrigated plots (see Table1). Thus, despite only making up 21% of total basal area, irrigated piñon transpiration comprised upwards of 50% of total *E*_C_ in irrigated plots (Fig.9, panel B) comprised of trees with a sufficiently high level of hydraulic function (i.e., high *k*_s_). This observation suggests that extensive piñon mortality in pinon–juniper woodlands due to drought will potentially have a more disproportionate impact on landscape-level carbon fixation and long-term carbon storage potential than simple stem density or basal area reductions alone might imply, even in piñon–juniper landscapes with lower piñon density. In contrast to irrigated plots, drought plots with low *k*_s_ exhibited *E*_C_ rates that were only 44% and 32% of *E*_C_ observed in ambient plots in years 2008 and 2012 (Fig.9, panel B). Compared to ambient plots, lower drought plot *E*_C_ rates in year 2008 were mainly driven by reduced precipitation (45% reduction from ambient) and lower plant *k*_s_ (as initial stem sapwood area in ambient and drought plots was quite similar in year 2007), whereas low *E*_C_ in drought plots in 2012 reflects the cumulative effects of reduced precipitation, prolonged reductions in plant *k*_s_, and loss of woody canopy to mortality (Table1, Figs8 and 9). In the absence of competitive release and/or compensation from nonwoody species, the long-term consequences of such productivity reductions in droughted plots are profound – with recovery of physiological and canopy function to predrought levels unlikely in the near term given the degree of canopy reduction (mortality) and the reductions of hydraulic function in surviving trees (not discounting new growth and xylem repair).

In summary, our results demonstrate how physiological responses to drought influence the susceptibility of piñon and juniper trees to drought-induced mortality, and how reduced hydraulic function and mortality directly affect landscape-level processes (i.e., total canopy *E*_C_ and *G*_C_) that directly link to net carbon uptake rates and potential carbon storage at the landscape scale. Given recent model projections of increasing temperature and reduced precipitation in the SWUS (Seager et al. 2007; Cayan et al. 2010), our results indicate that reductions in plant hydraulic conductance, decreased net carbon assimilation, and increased mortality can be expected for both piñon pine and juniper under anticipated future conditions of more frequent and persistent regional drought (Williams et al. 2013). Such reductions in productivity and woody plant cover in piñon–juniper woodlands will have negative impacts on regional carbon stocks (Schwalm et al. 2012; Jiang et al. 2013), as piñon–juniper woodlands have been observed to have higher annual productivity, net carbon uptake rates, and total ecosystem carbon storage compared to the dryer grassland and desert shrub land vegetation types that exist in this semiarid and dryland region (Anderson-Teixeira et al. 2011).

## Supporting Information

**Figure S1.** Relationship between nocturnal sap-flow output (Δ*T*_max_, *y*-axis) and nocturnal VPD (*x*-axis) for a 48 day period during conditions with higher VWC.

**Figure S2.** Radial pattern of xylem sap-flow (*J*_S_) with sapwood depth for both piñon and juniper over 180 days in 2011 and 2012.

**Figure S3.** Annual sap-flow (kg cm^−2^ year^−1^, plot mean) by species and treatment across the 2008 through 2012 period.

**Figure S4.** Stem diameter distribution across all plots for piñon and juniper in year 2007. Error bars (±1 SE) represent the variation between *n* = 12 plots (across all treatments).

**Figure S5.** Comparison of piñon sapwood area measured in year 2012 (open symbols, dashed regression line) versus sapwood area predicted (solid regression line) via the piñon allometric model that was developed in year 2007 (see Note S2).

**Figure S6.** Supplemental climate summary for the 5+ years period starting in 2007.

**Figure S7.** Midday foliar water potentials ( *Ψ*_MD_) for piñon (A) and juniper (B) across irrigation, ambient, and drought treatments during the 5+ year study period.

**Figure S8.** Midday sap-flow patterns (*J*_S_) for ambient and cover-control treatments across the 5+ year study period (panel A – piñon, panel B - juniper).

**Figure S9.** Relationship between *k*_s_ and predawn water potential (*Ψ*_PD_) across the 5+ year study period for each species and treatment (panel A).

**Figure S10.** Relationship between midday sap-flow (*J*_S_) and predawn water potential (*Ψ*_PD_) across the 5+ year period for each species and treatment (panel A).

**Note S1.** Calculation of sap-flux density using the Granier (1987) empirical relationship.

**Note S2.** Sapwood and leaf area allometric models for piñon and juniper.

**Table S1.** Nocturnal and mid-morning stomatal conductance (Leaf *g*_s_) rates for *P. edulis* and *J. monosperma* trees measured across three separate dates that varied in soil moisture content (VWC%, plot mean).

**Table S2**. ANCOVA model results for linear mixed model analysis of relationships between: 1) *k*_s_ versus predawn-Psi (*Ψ*_PD_), 2) midday leaf Δ*Ψ* versus *Ψ*_PD_, 3) midday *J*_S_ versus *Ψ*_PD_, and 4) midday *J*_S_ versus midday Δ*Ψ*.

**Table S3.** ANCOVA model results for analysis with transformed response variables. Linear mixed model analysis of relationships between: 1) *k*_s_ versus predawn-Psi (*Ψ*_PD_), 2) midday leaf Δ*Ψ* versus *Ψ*_PD_, 3) midday *J*_S_ versus *Ψ*_PD_, and 4) midday *J*_S_ versus midday Δ*Ψ*.
