# Supplementary material for: Prolonged experimental drought reduces plant hydraulic conductance and transpiration and increases mortality in a piñon–juniper woodland
Source: Ecol Evol. 2015 Mar 23;5(8):1618–38. doi: 10.1002/ece3.1422 (PMC4409411; doi:10.1002/ece3.1422)

Supplemental - Figure S1. Relationship between nocturnal sap-flow output (ΔT max, y-axis) and nocturnal *VPD* (x-axis) for a 48 day period during conditions with higher *VWC*. Each probe (per species) exhibits the typical pattern seen in the overwhelming majority of sensors.

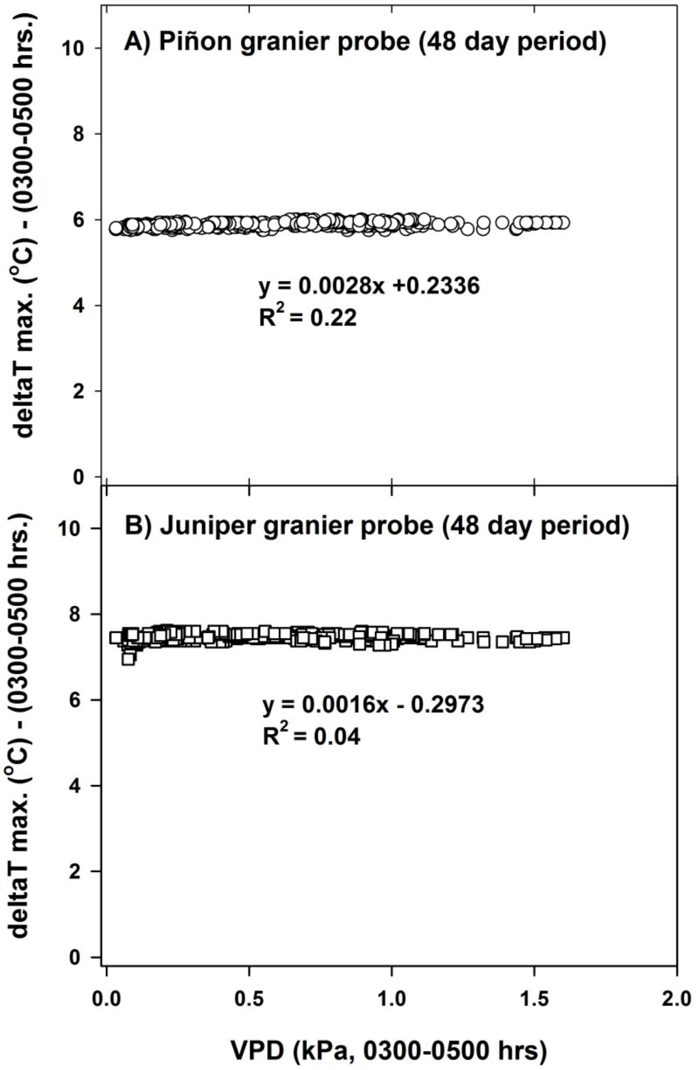

Supplement: Supplementary file 1 [file ece30005-1618-sd1.pdf]
