# Supplementary material for: Prolonged experimental drought reduces plant hydraulic conductance and transpiration and increases mortality in a piñon–juniper woodland
Source: Ecol Evol. 2015 Mar 23;5(8):1618–38. doi: 10.1002/ece3.1422 (PMC4409411; doi:10.1002/ece3.1422)

Supplemental - Figure S2. Radial pattern of xylem sap-flow (J_S) with sapwood depth for both piñon and juniper over 180 days in 2011 and 2012. Comparisons are made between sap-flow rates at A) 0-10 mm and 10-20 mm depth in piñon, B) 0-10 mm and 20-30 mm depth in piñon, and C) 0-10 mm and 10-20 mm depth in juniper. A total of n=4 piñon and n=3 juniper trees were measured in this analysis.

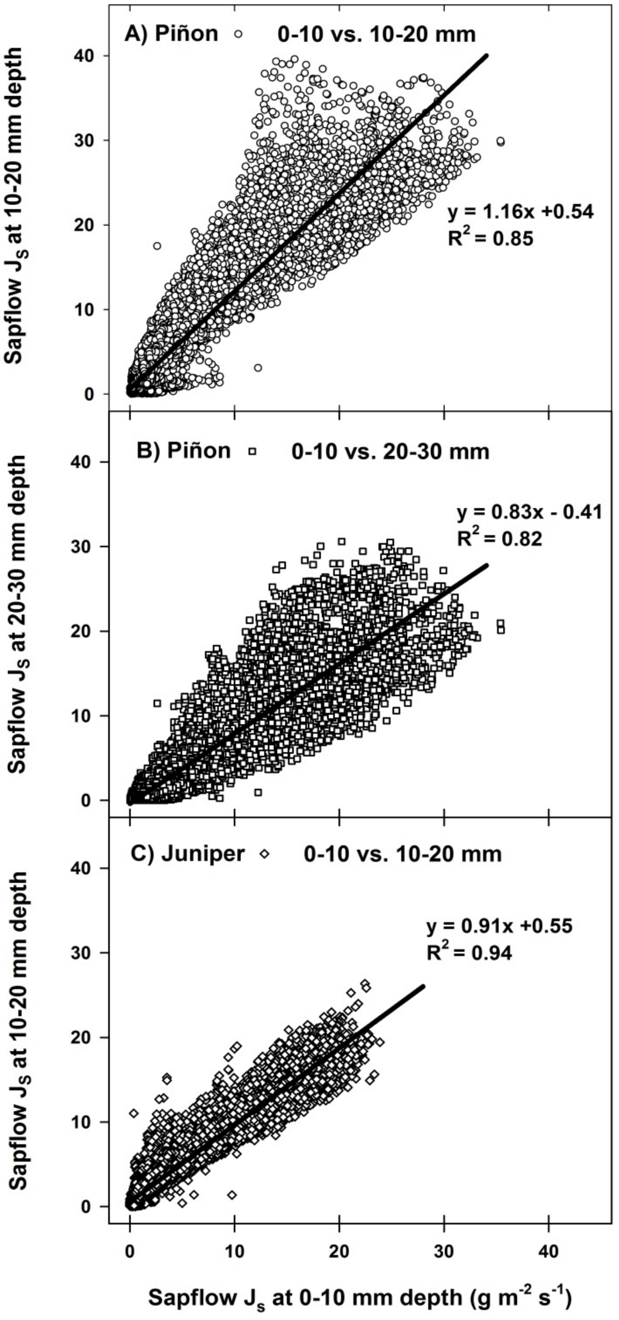

Supplement: Supplementary file 2 [file ece30005-1618-sd2.pdf]
