# Supplementary material for: Prolonged experimental drought reduces plant hydraulic conductance and transpiration and increases mortality in a piñon–juniper woodland
Source: Ecol Evol. 2015 Mar 23;5(8):1618–38. doi: 10.1002/ece3.1422 (PMC4409411; doi:10.1002/ece3.1422)

Supplemental - Figure S3. Annual sap-flow (kg cm^{-2} yr^{-1}, plot mean) by species and treatment across the 2008 through 2012 period. Annual sap-flow estimates were based on measurements from one replicate block (n=1) in years 2008 and 2009, and all replicate blocks (n=3) in years 2010 through 2012. For years 2010-2012, error bars (± 1 S.E.) represent the variation between plot means (by treatment).

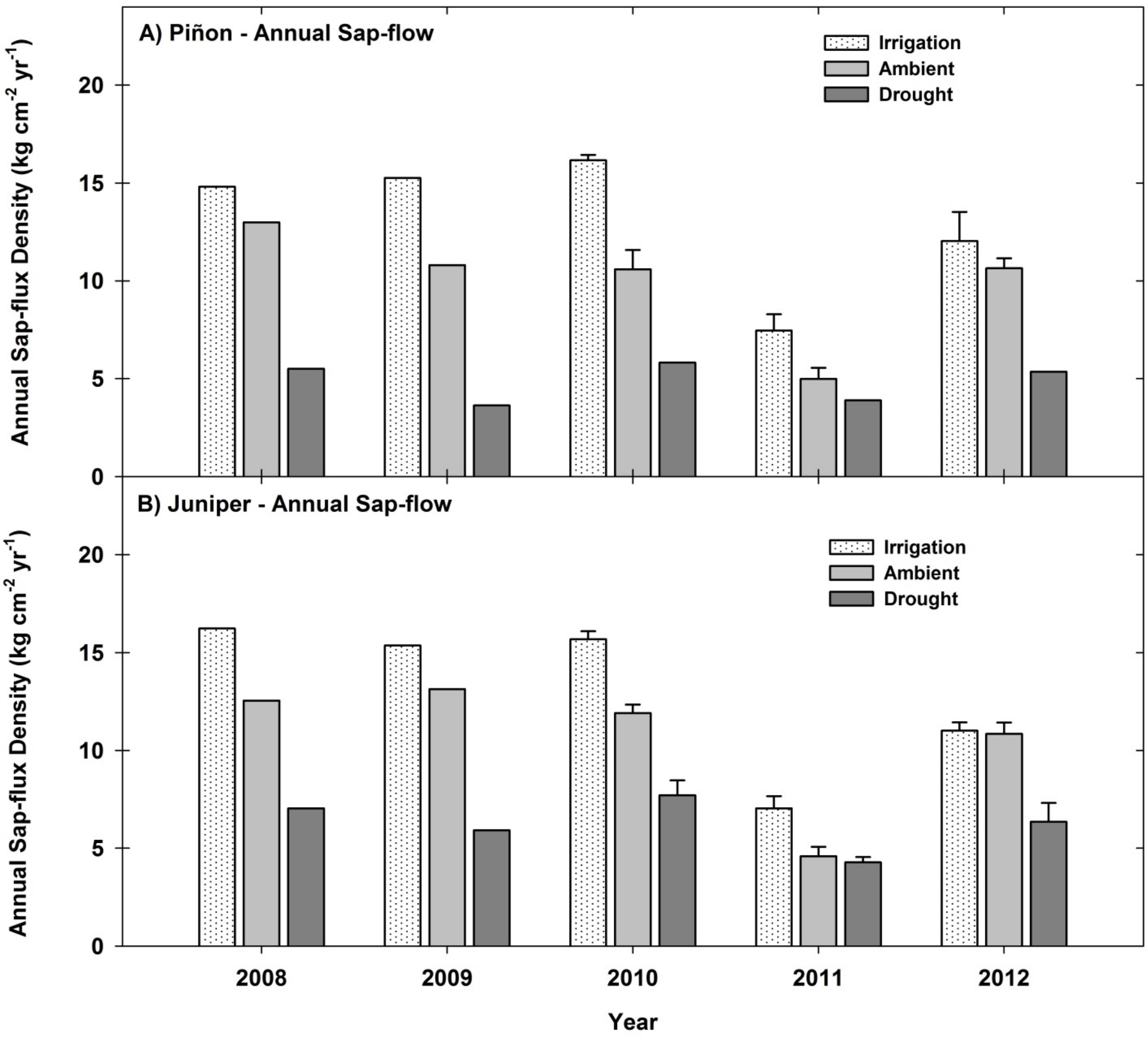

Supplement: Supplementary file 3 [file ece30005-1618-sd3.pdf]
