# Supplementary material for: Prolonged experimental drought reduces plant hydraulic conductance and transpiration and increases mortality in a piñon–juniper woodland
Source: Ecol Evol. 2015 Mar 23;5(8):1618–38. doi: 10.1002/ece3.1422 (PMC4409411; doi:10.1002/ece3.1422)

Supplemental - Figure S4. Stem diameter distribution across all plots for piñon and juniper in year 2007. Error bars (± 1 S.E.) represent the variation between n=12 plots (across all treatments). Values are the count per hectare by 5 cm diameter classes (for example, the 15 cm class represents all stems ranging from 10.0 to 14.99 cm in diameter). Stems were measured and counted down to a minimum stem diameter of 0.5 cm for the 0 to 4.99 cm diameter class.

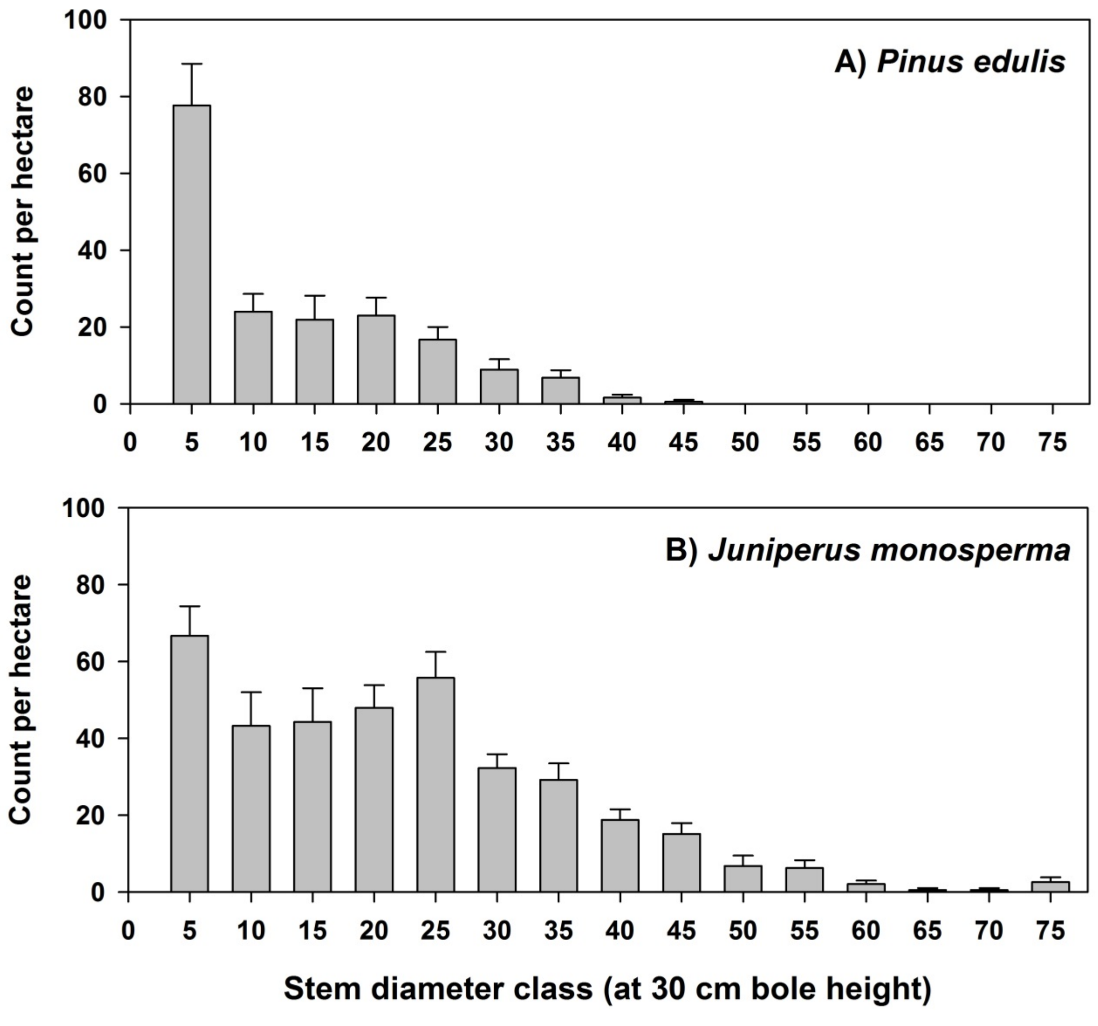

Supplement: Supplementary file 4 [file ece30005-1618-sd4.pdf]
