# Supplementary material for: Prolonged experimental drought reduces plant hydraulic conductance and transpiration and increases mortality in a piñon–juniper woodland
Source: Ecol Evol. 2015 Mar 23;5(8):1618–38. doi: 10.1002/ece3.1422 (PMC4409411; doi:10.1002/ece3.1422)

Supplemental - Figure S5. Comparison of piñon sapwood area measured in year 2012 (open symbols, dashed regression line) versus sapwood area predicted (solid regression line) via the piñon allometric model that was developed in year 2007 (see Suppl. Note S2). For the 2012 sampling, increment cores were taken from the main stem and two large branches from sap-flow instrumented trees (n=5 trees per treatment) growing in the flat aspect replicate block (Plots 1, 2, & 4). Sapwood area was determined using measurements of sapwood depth (determined from increment cores) and diameter.

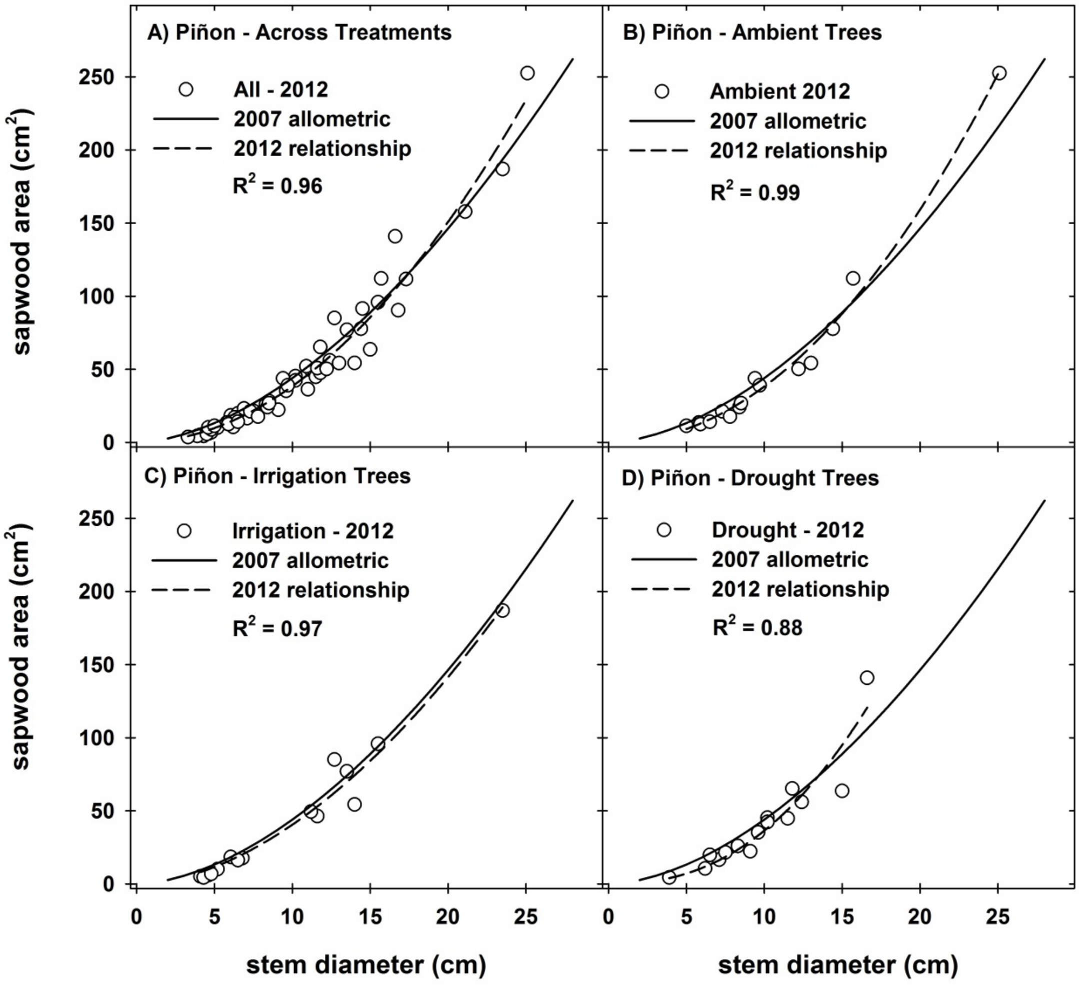

Supplement: Supplementary file 5 [file ece30005-1618-sd5.pdf]
