# Supplementary material for: Prolonged experimental drought reduces plant hydraulic conductance and transpiration and increases mortality in a piñon–juniper woodland
Source: Ecol Evol. 2015 Mar 23;5(8):1618–38. doi: 10.1002/ece3.1422 (PMC4409411; doi:10.1002/ece3.1422)

Supplemental - Figure S6. Supplemental climate summary for the 5+ yr period starting in 2007. A) Daily maximum *VPD* across 5+ yr period, B) Daily minimum *VPD*, C) Daily maximum *PAR*, D) Daily maximum temperature, E) Daily minimum temperature, and F) Daily mean wind speed.

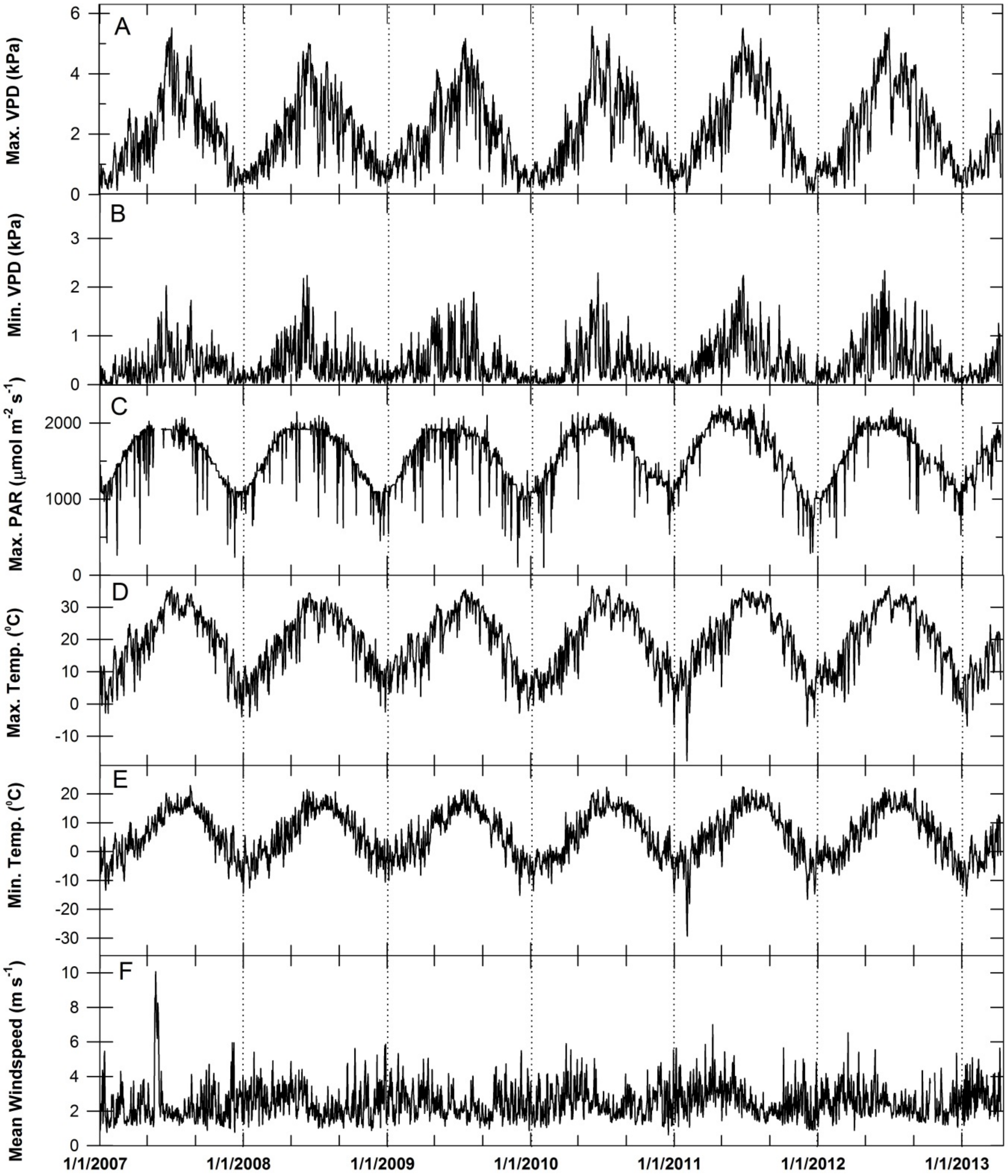

Supplement: Supplementary file 6 [file ece30005-1618-sd6.pdf]
