# Supplementary material for: Prolonged experimental drought reduces plant hydraulic conductance and transpiration and increases mortality in a piñon–juniper woodland
Source: Ecol Evol. 2015 Mar 23;5(8):1618–38. doi: 10.1002/ece3.1422 (PMC4409411; doi:10.1002/ece3.1422)

Supplemental - Figure S7. Midday foliar water potentials (Ψ_{MD}) for piñon (A) and juniper (B) across irrigation, ambient, and drought treatments during the 5+ yr study period. Means are ±1 S.E. Horizontal lines indicate 5+ yr treatment mean for each species as follows: irrigation (— — —), ambient (———), and drought (— ¨ — ¨ —).

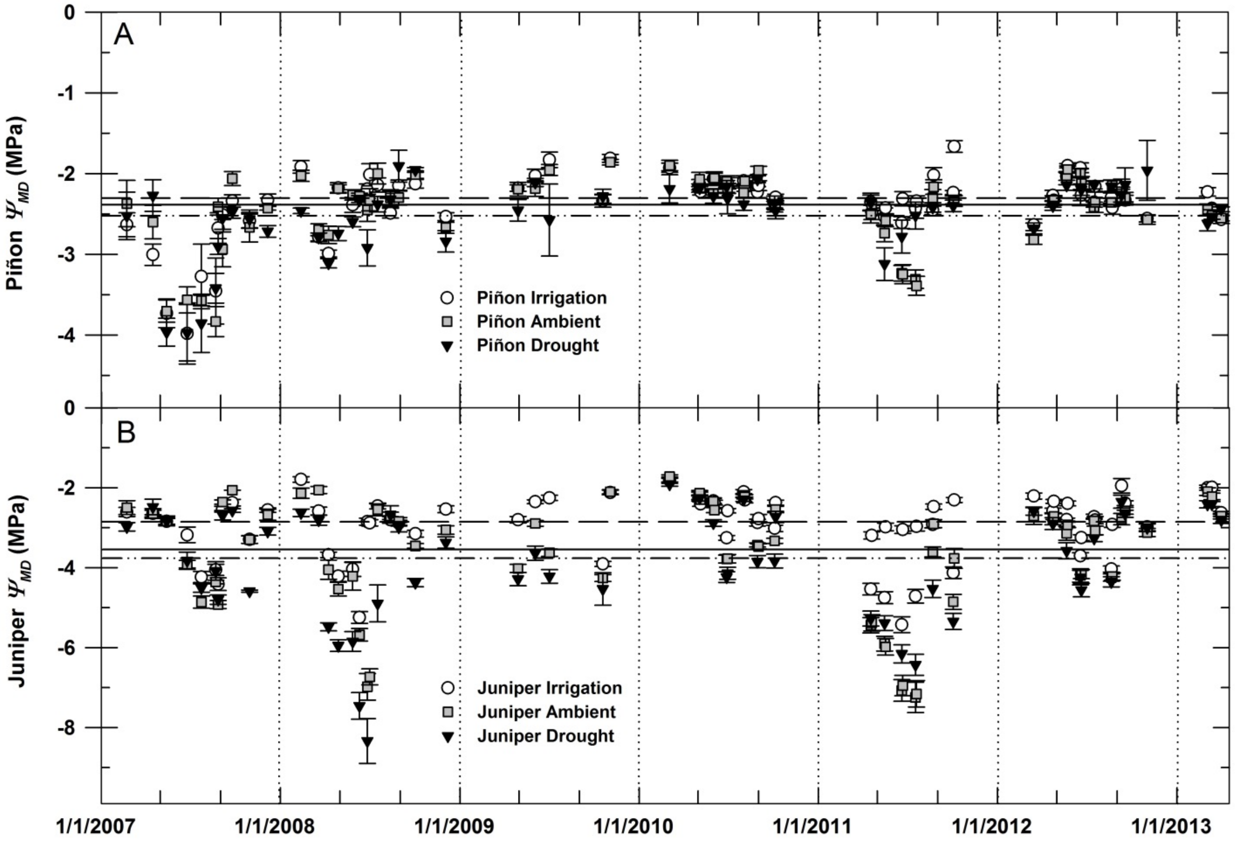

Supplement: Supplementary file 7 [file ece30005-1618-sd7.pdf]
