# Supplementary material for: Prolonged experimental drought reduces plant hydraulic conductance and transpiration and increases mortality in a piñon–juniper woodland
Source: Ecol Evol. 2015 Mar 23;5(8):1618–38. doi: 10.1002/ece3.1422 (PMC4409411; doi:10.1002/ece3.1422)

Supplemental - Figure S8. Midday sap-flow patterns (J_S) for ambient and cover-control treatments across the 5+ yr study period (panel A – piñon, panel B - juniper). For each year, data is the daily mean midday J_S rate (1100 to 1400 hrs.) across all days from March to October. Cover-control treatments were fully implemented in August 2007 (downward arrow). Error bars were not included to preserve clarity and view-ability of the plots. Midday J_S error estimates for ambient means are provided in Suppl. Fig. S10 regression plots.

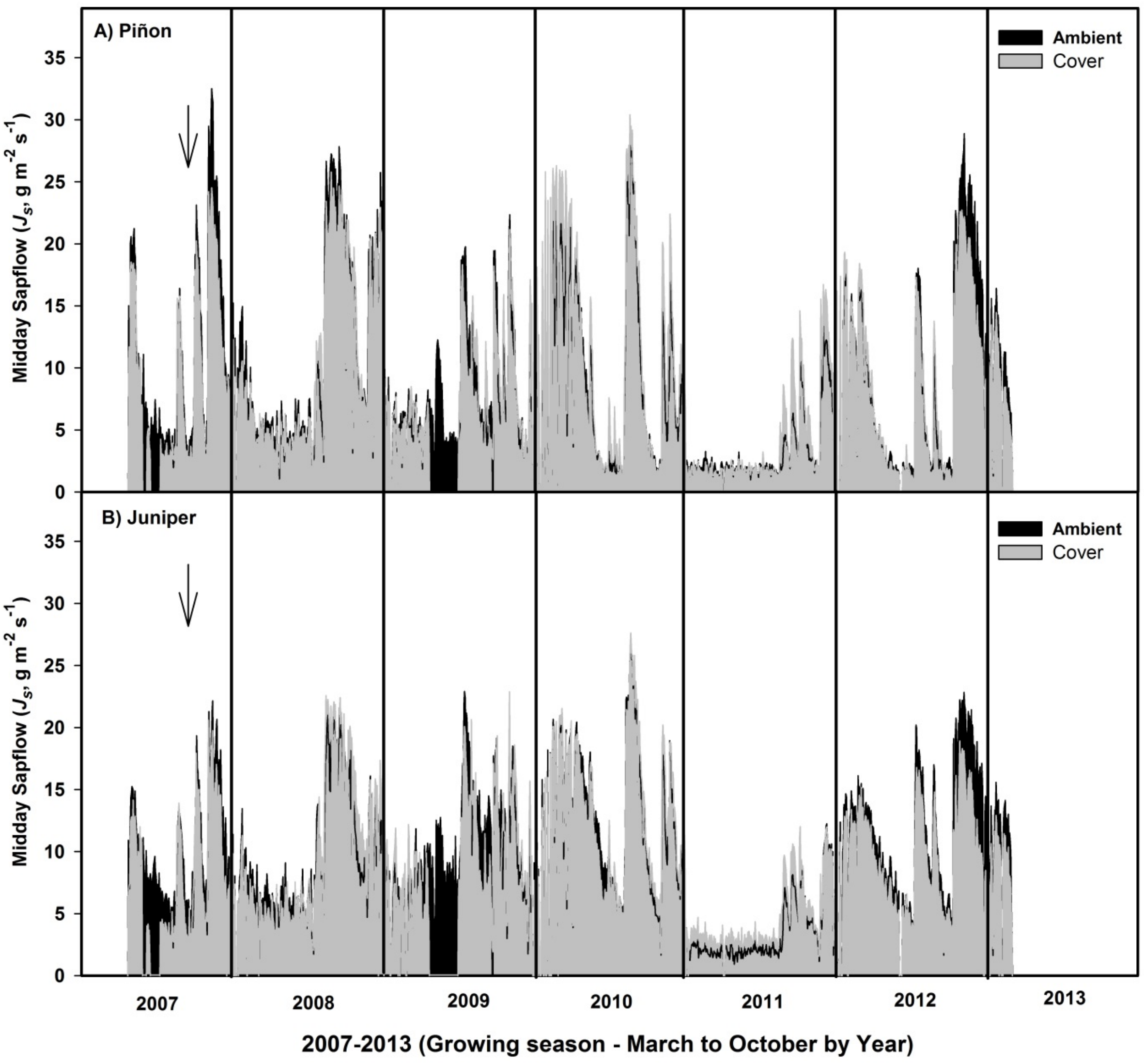

Supplement: Supplementary file 8 [file ece30005-1618-sd8.pdf]
