# Supplementary material for: Prolonged experimental drought reduces plant hydraulic conductance and transpiration and increases mortality in a piñon–juniper woodland
Source: Ecol Evol. 2015 Mar 23;5(8):1618–38. doi: 10.1002/ece3.1422 (PMC4409411; doi:10.1002/ece3.1422)

**Supplemental - Figure S9.** Relationship between $k_s$ and predawn water potential ( $\Psi_{PD}$) across the 5+ yr study period for each species and treatment (panel A). Response of $\Delta\Psi$ ($\Psi_{PD}$- $\Psi_{MD}$) to plant water stress ( $\Psi_{PD}$) across the study period (panel B). Values are mean ± 1 S.E. Regression fits are the best fit relationship using a two parameter exponential function of the form; $y = ae^{bx}$.

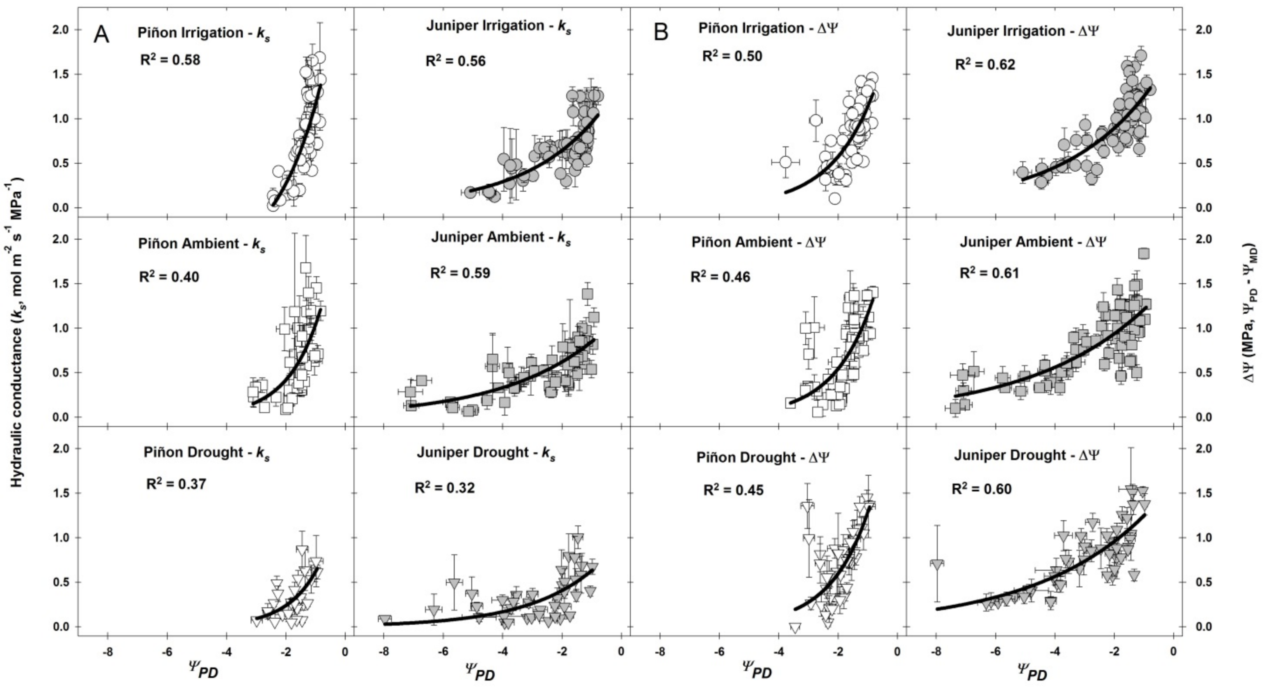

Supplement: Supplementary file 9 [file ece30005-1618-sd9.pdf]
