# Supplementary material for: Prolonged experimental drought reduces plant hydraulic conductance and transpiration and increases mortality in a piñon–juniper woodland
Source: Ecol Evol. 2015 Mar 23;5(8):1618–38. doi: 10.1002/ece3.1422 (PMC4409411; doi:10.1002/ece3.1422)

Supplemental - Figure S10. Relationship between midday sap-flow (J_S) and predawn water potential (Ψ_{PD}) across the 5+ yr period for each species and treatment (panel A). Response of midday J_S to $\Delta\Psi$ (Ψ_{PD}- Ψ_{MD}) across the study period (panel B). Values are mean ± 1 S.E. Non-linear regression fits are the best fit relationship using a two parameter exponential function of the form; $y = ae^{bx}$.

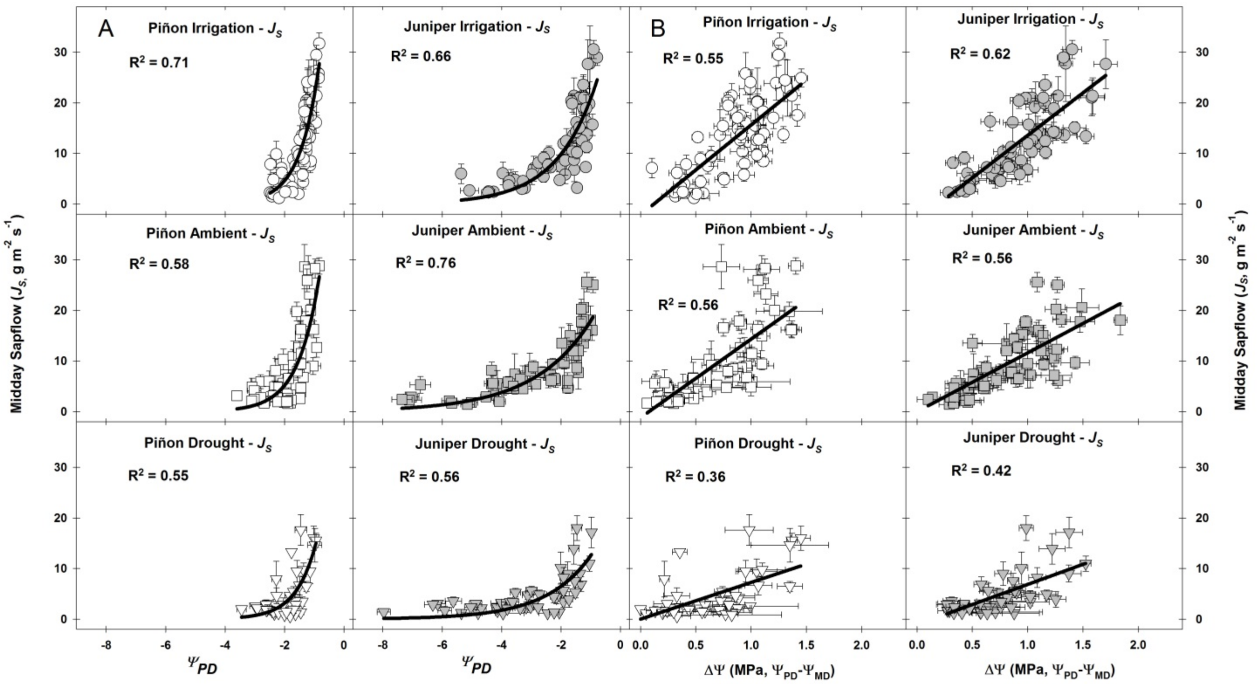

Supplement: Supplementary file 10 [file ece30005-1618-sd10.pdf]
