# Supplementary material for: Prolonged experimental drought reduces plant hydraulic conductance and transpiration and increases mortality in a piñon–juniper woodland
Source: Ecol Evol. 2015 Mar 23;5(8):1618–38. doi: 10.1002/ece3.1422 (PMC4409411; doi:10.1002/ece3.1422)
Supplement: Supplementary file 11 [file ece30005-1618-sd11.pdf]

Supplemental Note S1.

The calculation of sap-flux density using the empirical relationship developed by Granier (1987) requires that a measurement of the maximum daily temperature difference between heated and unheated reference probes (i.e., ΔT max or "dTmax") be made under conditions of zero water flux in the stem (which generally occurs at night, when stomata are closed and *VPD* is low). This is a critical condition that should be met, as calculated estimates of diurnal J_S (sap-flux density) will be underestimated if significant flux is present in the stem at night. Nocturnal transpiration due to partly open stomata has been observed in a number of species (Caird et al. 2007; Dawson et al. 2007; Marks and Lechowicz 2007), and significant nocturnal water flow in the stem due to transpiration would preclude the required conditions of zero flux needed to accurately determine dTmax values. We made multiple measurements of nocturnal conductance and transpiration using an LI-1600 porometer (LI-COR, Inc., Lincoln, NE, USA) under both pre-monsoon and monsoon conditions to assess if nocturnal transpiration and non-zero flow conditions at night were an issue at our site. We determined that under drought (low *VWC*) conditions stomata were fully closed at night (< 3.0 mmol m^{-2} s^{-1}) in both species examined in our study (see Suppl. Table S1). Under conditions of higher *VWC* during the monsoon season, we observed very low rates of nocturnal conductance (~ 4-7 mmol m^{-2} s^{-1}) in both species, and these non-zero nocturnal rates averaged approximately 6-7% of the daytime maximum rates observed (see monsoon period, Suppl. Table S1). To further assess if these conditions of non-zero nocturnal conductance affected our nocturnal sap-flow rates, we plotted daily sap-flow rates observed during the hours of 0300 to 0500 hrs (y-axis) against nocturnal *VPD* (x-axis) for a 48-day period (wet period, with high *VWC*) to determine if any relationship existed between these two variables. In this analysis, if nocturnal flux in stem were present - a negative relationship

should exist between the two variables, with nights at higher *VPD* conditions (0300-0500 hrs) having lower "dTmax" values. These lower "dTmax" values would be driven by decreases in temperature differences between the heated and unheated reference sap-flow probes due to increased heat dissipation caused by significant water flow in the stem. We plotted individual relationships for a number of probes for both piñon and juniper in one of our irrigation plots, and in no instances did we observe a negative relationship between nocturnal "dTmax" observations and *VPD* (for an example of the typical relationship we observed in both species, see Suppl. Fig. S1). Thus, for the purposes of using the Grainer sap-flow method, we concluded that the assumption of zero nocturnal stem flux (at 0300-0500 hrs) was justified for the two species at our site. Additionally, the observation of zero to very low nocturnal conductance for the species at our site (Suppl. Table S1) allowed us to assume that disequilibrium between foliar Ψ_{PD} and soil water potential (Ψ_S) due to nocturnal transpiration was negligible (see Donovan et al. 1999; Donovan et al. 2003; and Kavanagh et al. 2007 for discussing of phenomenon).

Supplemental Note S1 – References.

Caird MA, Richards JH, Donovan LA (2007) Nighttime Stomatal Conductance and Transpiration in C_3 and C_4 Plants. Plant Physiology 143, 4–10.

Dawson TE, Burgess S, Tu KP et al. (2007) Nighttime transpiration in woody plants from contrasting ecosystems. Tree Physiology 27, 561–575.

Donovan LA, Grisé DJ, West JB, Pappert RA, Alder NN, Richards JH (1999) Predawn disequilibrium between plant and soil water potentials in two cold-desert shrubs. Oecologia 120, 209-217.

Donovan, LA, Richards JH, Linton MJ (2003) Magnitude and mechanisms of disequilibrium between predawn plant and soil water potentials. Ecology 84, 463–470.

Granier, A (1987) Evaluation of transpiration in a Douglas-fir stand by means of sap-flow measurements. Tree Physiology 3, 309–320.

Kavanagh KL, Pangle R, Schotzko AD (2007) Nocturnal transpiration causing disequilibrium between soil and stem predawn water potential in mixed conifer forests of Idaho. Tree Physiology 27, 621–629.

Marks CO, Lechowicz MJ (2007) The ecological and functional correlates of nocturnal transpiration. Tree Physiology 27, 577–584.
