# Supplementary material for: Prolonged experimental drought reduces plant hydraulic conductance and transpiration and increases mortality in a piñon–juniper woodland
Source: Ecol Evol. 2015 Mar 23;5(8):1618–38. doi: 10.1002/ece3.1422 (PMC4409411; doi:10.1002/ece3.1422)
Supplement: Supplementary file 12 [file ece30005-1618-sd12.pdf]

Supplemental Note S2.

Sapwood and leaf area allometric models for piñon and juniper trees developed in year 2007 from destructively harvested branches and/or small stems sampled from off plot trees growing within 1 km of the study site. Destructively harvested branches used to develop allometric relationships ranged in diameter from 1.8 to 15.9 cm for piñon (n=16 samples), and from 1.7 to 18.6 cm for juniper (n=24 samples).

Allometric models (x = stem diameter, cm)
model form; $y = ax^b$

**Piñon models**	**Sapwood model**	**Leaf area model**
coefficients	sapwood (cm^2)	leaf area (m^2)
a	0.8112	0.0889
b	1.7341	1.9172
model fit	R^2 = 0.9066	R^2 = 0.9687

**Juniper models**	**Sapwood model**	**Leaf area model**
coefficients	sapwood (cm^2)	leaf area (m^2)
a	0.8227	0.0773
b	1.3903	1.686
model fit	R^2 = 0.8588	R^2 = 0.9148
