# Supplementary material for: Prolonged experimental drought reduces plant hydraulic conductance and transpiration and increases mortality in a piñon–juniper woodland
Source: Ecol Evol. 2015 Mar 23;5(8):1618–38. doi: 10.1002/ece3.1422 (PMC4409411; doi:10.1002/ece3.1422)
Supplement: Supplementary file 13 [file ece30005-1618-sd13.pdf]

Supplemental Table S1. Nocturnal and mid-morning stomatal conductance (Leaf g_s) rates for *P. edulis* and *J. monosperma* trees measured across three separate dates that varied in soil moisture content (*VWC*%, plot mean). Leaf g_s was measured with a LI-1600 porometer (Licor, Inc.) during both pre-monsoon and monsoon periods in irrigated, drought, and ambient treatment plots. Values are means +/- 1 S.E. n=5 trees per treatment were measured on each date.

Species & Treatment	8/12/2010 Leaf g_s (mmol m^{-2} s^{-1}) monsoon season w/o irrigation (n=5)			6/23/2011 Leaf g_s (mmol m^{-2} s^{-1}) pre-monsoon w/ irrigation (n=5)			6/18/2012 Leaf g_s (mmol m^{-2} s^{-1}) pre-monsoon w/o irrigation (n=5)		
Period→	8-11am	1-5am	*VWC*	8-11am	1-5am	*VWC*	8-11am	1-5am	*VWC*
Piñon (Irrigation)	112.3 (19.6)	7.2 (2.6)	10.4% (0.9)	35.3 (3.2)	2.4 (0.7)	8.5% (1.0)	5.6 (1.4)	1.2 (0.4)	3.7% (0.6)
Piñon (Drought)	62.9 (13.3)	2.6 (0.8)	5.4% (0.5)	2.3 (0.8)	0.5 (0.2)	< 2%	4.7 (0.7)	0.6 (0.3)	2.7% (0.3)
Piñon (Ambient)	n/a	n/a	n/a	n/a	n/a	n/a	6.2 (0.8)	1.5 (0.5)	3.8% (0.8)
Juniper (Irrigation)	92.2 (4.5)	6.5 (2.0)	10.4% (0.9)	38.8 (4.1)	5.3 (1.4)	8.5% (1.0)	13.0 (1.7)	1.7 (0.5)	3.7% (0.6)
Juniper (Drought)	60.8 (10.7)	4.6 (0.6)	5.4% (0.5)	7.4 (1.8)	0.4 (0.1)	< 2%	13.6 (1.7)	1.2 (0.3)	2.7% (0.3)
Juniper (Ambient)	n/a	n/a	n/a	n/a	n/a	n/a	19.1 (1.2)	2.9 (0.2)	3.8% (0.8)
