# Supplementary material for: Prolonged experimental drought reduces plant hydraulic conductance and transpiration and increases mortality in a piñon–juniper woodland
Source: Ecol Evol. 2015 Mar 23;5(8):1618–38. doi: 10.1002/ece3.1422 (PMC4409411; doi:10.1002/ece3.1422)
Supplement: Supplementary file 14 [file ece30005-1618-sd14.pdf]

Supplemental Table S2. ANCOVA model results for linear mixed model analysis of relationships between: 1) k_s vs. predawn-Psi (Ψ_{PD}), 2) midday leaf $\Delta\Psi$ vs. Ψ_{PD}, 3) midday J_S vs. Ψ_{PD}, and 4) midday J_S vs. midday $\Delta\Psi$. *Significant differences for ambient treatments indicate that intercepts and slopes are significantly different from zero. [a]Significant differences for irrigation and drought treatment indicate a significant difference from ambient treatment model estimates for a specific parameter. Significant differences at a threshold value of $p \leq 0.05$ are indicated in bold text.

Species	Treatment	Transformation Type	Predictor Variable (x)	Response Variable (y)	Intercept-Est. & (SE)	Slope-Est. & (SE)	Intercept Pr > |t| & t-value	Slope Pr > |t| & t-value
Piñon	Irrigation	none	Ψ_{PD}	k_s	1.438 (0.118)	0.468 (0.080)	**p=0.0082[a] (2.65)**	**p=0.0344[a] (2.12)**
Piñon	Ambient	none	Ψ_{PD}	k_s	1.126 (0.083)	0.298 (0.052)	**p<0.0001[*] (13.5)**	**p<0.0001[*] (5.68)**
Piñon	Drought	none	Ψ_{PD}	k_s	0.610 (0.184)	0.158 (0.104)	**p=0.0050[a] (-2.81)**	p=0.1806 (-1.34)
Juniper	Irrigation	none	Ψ_{PD}	k_s	1.094 (0.061)	0.210 (0.024)	**p=0.0012[a] (3.26)**	**p=0.0008[a] (3.36)**
Juniper	Ambient	none	Ψ_{PD}	k_s	0.896 (0.044)	0.131 (0.015)	**p<0.0001[*] (20.4)**	**p<0.0001[*] (9.07)**
Juniper	Drought	none	Ψ_{PD}	k_s	0.589 (0.071)	0.091 (0.024)	**p<0.0001[a] (-4.33)**	p=0.0870 (-1.71)
Piñon	Irrigation	none	Ψ_{PD}	$\Delta\Psi$	1.695 (0.063)	0.580 (0.038)	p=0.4981 (0.68)	p=0.5223 (0.64)
Piñon	Ambient	none	Ψ_{PD}	$\Delta\Psi$	1.652 (0.046)	0.556 (0.025)	**p<0.0001[*] (35.6)**	**p<0.0001[*] (22.0)**
Piñon	Drought	none	Ψ_{PD}	$\Delta\Psi$	1.510 (0.102)	0.446 (0.052)	p=0.1606 (-1.40)	**p=0.0353[a] (-2.11)**
Juniper	Irrigation	none	Ψ_{PD}	$\Delta\Psi$	1.475 (0.045)	0.266 (0.017)	**p=0.0238[a] (2.26)**	**p<0.0001[a] (3.92)**
Juniper	Ambient	none	Ψ_{PD}	$\Delta\Psi$	1.374 (0.032)	0.199 (0.010)	**p<0.0001[*] (43.3)**	**p<0.0001[*] (19.5)**
Juniper	Drought	none	Ψ_{PD}	$\Delta\Psi$	1.280 (0.049)	0.176 (0.015)	p=0.0557 (-1.92)	p=0.1188 (-1.56)
Piñon	Irrigation	none	Ψ_{PD}	midday J_S	24.97 (1.144)	8.242 (0.596)	**p=0.0021[a] (3.08)**	p=0.0553 (1.92)
Piñon	Ambient	none	Ψ_{PD}	midday J_S	21.45 (0.872)	7.099 (0.421)	**p<0.0001[*] (24.6)**	**p<0.0001[*] (16.9)**
Piñon	Drought	none	Ψ_{PD}	midday J_S	10.49 (1.856)	3.220 (0.831)	**p<0.0001[a] (-5.90)**	**p<0.0001[a] (-4.67)**
Juniper	Irrigation	none	Ψ_{PD}	midday J_S	20.12 (0.834)	4.008 (0.221)	**p<0.0001[a] (4.68)**	**p<0.0001[a] (7.12)**
Juniper	Ambient	none	Ψ_{PD}	midday J_S	16.22 (0.611)	2.435 (0.140)	**p<0.0001[*] (26.5)**	**p<0.0001[*] (17.4)**
Juniper	Drought	none	Ψ_{PD}	midday J_S	8.880 (0.921)	1.231 (0.212)	**p<0.0001[a] (-7.96)**	**p<0.0001[a] (-5.67)**
Piñon	Irrigation	none	$\Delta\Psi$	midday J_S	4.362 (0.910)	10.31 (0.872)	**p<0.0261[a] (2.23)**	p=0.4391 (0.77)
Piñon	Ambient	none	$\Delta\Psi$	midday J_S	2.336 (0.585)	9.633 (0.595)	**p<0.0001[*] (4.00)**	**p<0.0001[*] (16.2)**
Piñon	Drought	none	$\Delta\Psi$	midday J_S	1.083 (1.220)	4.666 (1.330)	p=0.3045 (-1.03)	**p=0.0002[a] (-3.73)**
Juniper	Irrigation	none	$\Delta\Psi$	midday J_S	5.289 (0.939)	7.497 (0.718)	p=0.9963 (-0.00)	**p=0.0002[a] (3.72)**
Juniper	Ambient	none	$\Delta\Psi$	midday J_S	5.293 (0.642)	4.824 (0.485)	**p<0.0001[*] (8.24)**	**p<0.0001[*] (9.96)**
Juniper	Drought	none	$\Delta\Psi$	midday J_S	2.693 (0.943)	3.134 (0.840)	**p=0.0059[a] (-2.76)**	**p=0.0443[a] (-2.01)**
