# Supplementary material for: Prolonged experimental drought reduces plant hydraulic conductance and transpiration and increases mortality in a piñon–juniper woodland
Source: Ecol Evol. 2015 Mar 23;5(8):1618–38. doi: 10.1002/ece3.1422 (PMC4409411; doi:10.1002/ece3.1422)
Supplement: Supplementary file 15 [file ece30005-1618-sd15.pdf]

Supplemental Table S3. ANCOVA model results for analysis with transformed response variables. Linear mixed model analysis of relationships between: 1) k_s vs. predawn-Psi (Ψ_{PD}), 2) midday leaf $\Delta\Psi$ vs. Ψ_{PD}, 3) midday J_S vs. Ψ_{PD}, and 4) midday J_S vs. midday $\Delta\Psi$. *Significant differences for ambient treatments indicate that intercepts and slopes are significantly different from zero. [a]Significant differences for irrigation and drought treatment indicate a significant difference from ambient treatment model estimates for a specific parameter. Significant differences at a threshold value of $p \leq 0.05$ are indicated in bold text.

Species	Treatment	Transformation Type	Predictor Variable (x)	Response Variable (y)	Intercept-Est. & (SE)	Slope-Est. & (SE)	Intercept Pr > |t| & t-value	Slope Pr > |t| & t-value
Piñon	Irrigation	ln(y+1)	Ψ_{PD}	k_s	0.944 (0.063)	0.294 (0.043)	**p=0.0086[a] (2.63)**	**p=0.0297[a] (2.18)**
Piñon	Ambient	ln(y+1)	Ψ_{PD}	k_s	0.778 (0.045)	0.201 (0.028)	**p<0.0001* (17.3)**	**p<0.0001* (7.14)**
Piñon	Drought	ln(y+1)	Ψ_{PD}	k_s	0.472 (0.098)	0.119 (0.056)	**p=0.0019[a] (-3.12)**	p=0.1388 (-1.48)
Juniper	Irrigation	ln(y+1)	Ψ_{PD}	k_s	0.716 (0.058)	0.121 (0.012)	**p=0.0481[a] (2.01)**	**p=0.0003[a] (3.66)**
Juniper	Ambient	ln(y+1)	Ψ_{PD}	k_s	0.599 (0.041)	0.076 (0.007)	**p<0.0001* (14.5)**	**p<0.0001* (10.3)**
Juniper	Drought	ln(y+1)	Ψ_{PD}	k_s	0.408 (0.058)	0.055 (0.012)	**p=0.0012[a] (-3.32)**	p=0.0776 (-1.77)
Piñon	Irrigation	ln(y+1)	Ψ_{PD}	$\Delta\Psi$	1.085 (0.037)	0.343 (0.022)	p=0.5127 (0.65)	p=0.6250 (0.49)
Piñon	Ambient	ln(y+1)	Ψ_{PD}	$\Delta\Psi$	1.061 (0.027)	0.333 (0.015)	**p<0.0001* (39.0)**	**p<0.0001* (22.7)**
Piñon	Drought	ln(y+1)	Ψ_{PD}	$\Delta\Psi$	0.978 (0.058)	0.270 (0.030)	p=0.1560 (-1.42)	**p=0.0355[a] (-2.10)**
Juniper	Irrigation	ln(y+1)	Ψ_{PD}	$\Delta\Psi$	0.939 (0.026)	0.152 (0.009)	**p=0.0157[a] (2.43)**	**p<0.0001[a] (4.45)**
Juniper	Ambient	ln(y+1)	Ψ_{PD}	$\Delta\Psi$	0.877 (0.018)	0.114 (0.005)	**p<0.0001* (48.3)**	**p<0.0001* (22.3)**
Juniper	Drought	ln(y+1)	Ψ_{PD}	$\Delta\Psi$	0.839 (0.027)	0.105 (0.008)	p=0.1668 (-1.39)	p=0.2192 (-1.23)
Piñon	Irrigation	sqrt(y)	Ψ_{PD}	midday J_S	5.273 (0.219)	1.355 (0.096)	**p=0.0336[a] (2.14)**	p=0.1984 (1.29)
Piñon	Ambient	sqrt(y)	Ψ_{PD}	midday J_S	4.805 (0.163)	1.232 (0.068)	**p<0.0001* (29.5)**	**p<0.0001* (18.1)**
Piñon	Drought	sqrt(y)	Ψ_{PD}	midday J_S	3.281 (0.335)	0.706 (0.135)	**p<0.0001[a] (-4.56)**	**p=0.0001[a] (-3.89)**
Juniper	Irrigation	sqrt(y)	Ψ_{PD}	midday J_S	4.557 (0.243)	0.677 (0.032)	**p=0.0014[a] (3.31)**	**p<0.0001[a] (9.84)**
Juniper	Ambient	sqrt(y)	Ψ_{PD}	midday J_S	3.751 (0.174)	0.358 (0.020)	**p<0.0001* (21.6)**	**p<0.0001* (17.8)**
Juniper	Drought	sqrt(y)	Ψ_{PD}	midday J_S	2.573 (0.235)	0.205 (0.031)	**p<0.0001[a] (-5.01)**	**p<0.0001[a] (-4.94)**
Piñon	Irrigation	sqrt(y)	$\Delta\Psi$	midday J_S	1.978 (0.196)	1.586 (0.131)	**p=0.0078[a] (2.73)**	p=0.2615 (-1.12)
Piñon	Ambient	sqrt(y)	$\Delta\Psi$	midday J_S	1.444 (0.132)	1.733 (0.089)	**p<0.0001* (10.9)**	**p<0.0001* (19.5)**
Piñon	Drought	sqrt(y)	$\Delta\Psi$	midday J_S	1.158 (0.238)	1.075 (0.201)	p=0.2327 (-1.20)	**p=0.0011[a] (-3.27)**
Juniper	Irrigation	sqrt(y)	$\Delta\Psi$	midday J_S	2.200 (0.266)	1.134 (0.103)	p=0.7709 (0.29)	**p=0.0008[a] (3.36)**
Juniper	Ambient	sqrt(y)	$\Delta\Psi$	midday J_S	2.123 (0.1871)	0.787 (0.069)	**p<0.0001* (11.4)**	**p<0.0001* (11.3)**
Juniper	Drought	sqrt(y)	$\Delta\Psi$	midday J_S	1.531 (0.250)	0.544 (0.120)	**p=0.0202[a] (-2.37)**	**p=0.0426[a] (-2.03)**
